# Alkaloid Extraction from *Coptis chinensis* Franch. Using Ultrasound-Assisted Aqueous Solutions of Surfactants, Organic Acids, Deep Eutectic Solvents, and Supramolecular Deep Eutectic Solvents

**DOI:** 10.3390/molecules30071418

**Published:** 2025-03-22

**Authors:** Khan Viet Nguyen, Nhan Trong Le, Vy Thao Thi Dang, Oleh Koshovyi, Ain Raal, Hoai Thi Nguyen

**Affiliations:** 1 Faculty of Pharmacy, Hue University of Medicine and Pharmacy, Hue University, Hue City 49000, Vietnam; nvkhan@hueuni.edu.vn (K.V.N.); ltrongnhan@hueuni.edu.vn (N.T.L.); dangthithaovy92@gmail.com (V.T.T.D.); 2 Institute of Pharmacy, Faculty of Medicine, University of Tartu, 50411 Tartu, Estonia; ain.raal@ut.ee

**Keywords:** protoberberine-type alkaloids, green solvents, carboxylic acids

## Abstract

Berberine, palmatine, and coptisine are bioactive alkaloids commonly found in medicinal plants, including *Coptis chinensis* Franch. (Ranunculaceae). To address the limitations of conventional volatile organic solvents, this study employed eco-friendly solvents—aqueous solutions of surfactants, carboxylic acids, and deep eutectic solvents—to extract these alkaloids. Among the solvents tested, lactic acid, malic acid, and pyruvic acid exhibited the highest extraction efficiencies. Optimal extraction conditions for ultrasound-assisted extraction were determined via response surface methodology. For lactic acid, optimal conditions included a concentration of 96% (*w*/*w*), a liquid-to-solid ratio of 30.0 mL/g, and a temperature of 60.0 °C, yielding 139.6 ± 0.2 mg/g of total alkaloids. Malic acid at 40.0% (*w*/*w*), 30.0 mL/g, and 80.0 °C produced 133.0 ± 0.5 mg/g, while pyruvic acid at 88.0% (*w*/*w*), 30.0 mL/g, and 75.0 °C resulted in 146.3 ± 0.4 mg/g. The recovery efficiencies of these alkaloids were further enhanced using macroporous resins. The XAD-8 and AB-8 resins achieved recovery rates of 80.11 ± 0.78% and 79.00 ± 1.06%, respectively, for lactic acid extracts. The LSA-40 resin yielded efficiencies of 95.58 ± 1.40% and 89.86 ± 0.90% for malic and pyruvic acid extracts, respectively. Notably, the combination of malic acid as an extraction solvent and the HPD-400 resin achieved an impressive alkaloid recovery yield of 79.52% from *C. chinensis*. This work represents the first reported application of this approach and highlights the potential of green solvents and macroporous resins for sustainable and efficient alkaloid extraction from *C. chinensis*.

## 1. Introduction

*Coptis chinensis* Franch. (Ranunculaceae), extensively utilized in traditional medicine, has a long history as a key ingredient in numerous traditional medicinal formulations [1,2]. *C. chinensis* exhibits properties including detoxification, heat clearing, purging fire, and dampness drying [3]. It has been used in medical practice to treat skin diseases, dysentery, diabetes, gastrointestinal infections, inflammation, and liver diseases [2,3]. Recent studies have shown that *C. chinensis* and its bioactive alkaloids exhibit a range of pharmacological activities. These include anti-tumor properties, with efficacy observed in various cancers, and antimicrobial effects against bacteria, viruses, fungi, protozoans, helminths, and chlamydia, as well as anti-inflammatory, antioxidant, anti-diabetic, lipid-lowering, and neuroprotective effects [1]. Among the various secondary metabolites of *C. chinensis*, alkaloids are the predominant components. Remarkably, protoberberine-type alkaloids, such as berberine, palmatine, and coptisine, are the principal bioactive constituents of *C. chinensis* [4].

Berberine, a quaternary benzylisoquinoline plant alkaloid, scientifically known as 5,6-dihydro-9,10-dimethoxybenzo[g]-1,3-benzodioxolo[5,6-a]quinolizinium, has been regarded as one of the most promising natural compounds for the treatment of various human diseases. Berberine is mainly found in the roots, rhizomes, and stem bark of many medicinal plants belonging to the families Ranunculaceae, Rutaceae, and Berberidaceae, such as *Coscinium fenestratum*, *Phellodendri amurensis*, and *C. chinensis* [5]. Berberine has been used in folk medicine since approximately 3000 BC, for its potent antimicrobial, antiprotozoal, antidiarrheal, and antitrachoma properties [6]. Over the years, clinical research on berberine has revealed a wide spectrum of pharmacological effects. Numerous studies have documented its significant antioxidant, anti-inflammatory, analgesic, anticancer, antihypertensive, antiarrhythmic, antihyperglycemic, antidepressant, anxiolytic, neuroprotective, antihypercholesterolemic, hypolipidemic, nephroprotective, hepatoprotective, cardioprotective, cerebroprotective, and antiviral activities [7,8]. In addition, berberine contributes significantly to the development of new pharmaceuticals by allowing modifications and substitutions at strategic positions to improve its desired biological properties [9]. Berberine has been and continues to be clinically evaluated for its significant therapeutic benefits and lower toxicity and side effects than synthetic drugs [10].

Palmatine, a protoberberine isoquinoline alkaloid with the chemical name 2,3,9,10-tetramethoxy-5,6-dihydroisoquinolino[2,1-b]isoquinolin-7-ium, has been extensively utilized in the pharmaceutical field [11]. Palmatine is a primary bioactive compound found in several botanical genera, such as *Berberis* spp., *Coptis* spp., *Phellodendron* spp., *Corydalis* spp., *Fibraurea* spp., *Papaver* spp., *Enantia* spp., and others [11]. Plants comprising palmatine have been employed in traditional medicine across numerous Asian countries for centuries, serving as treatments for a range of ailments, including jaundice, liver diseases, hypertension, inflammation, and dysentery [12]. Furthermore, palmatine possesses antioxidant, antibacterial, antiviral, anticancer, neuroprotective, and blood lipid regulation properties. Palmatine is valuable in preventing and treating various diseases, such as cancer, cardiac hypertrophy, osteoporosis, diabetes and its complications, Alzheimer’s disease, atopic dermatitis, osteoarthritis, and age-related conditions [11].

Coptisine, a benzyl tetrahydroisoquinoline alkaloid with the chemical name 7,8,13,13a-tetradehydro-2′H,2′′H-bis([1,3]dioxolo)[4′,5′:2,3;4′′,5′′:9,10]berbin-7-ium, has been employed in traditional medicine for thousands of years [13]. To date, numerous studies have reported the various pharmacological properties of coptisine. Coptisine has demonstrated potential as an anti-inflammatory, anticancer, coronary artery disease-ameliorating, antibacterial agent by modulating signal transduction pathways including NF-κB, NLRP3 inflammasome, MAPK, RANKL/RANK, PI3K/Akt, and Beclin 1/Sirt1; it has also exhibited neuroprotective activity by inhibiting the activation of microglia and astrocytes through the inactivation of CD11b and GFAP [2]. Furthermore, this compound exhibits significant pharmacological effects in myocardial ischemia and reperfusion damage, in gastric mucosa protection, in osteoclast differentiation inhibition, and against symptoms related to diabetic complications [14].

Considering the benefits of berberine, palmatine, and coptisine, extracting these compounds from medicinal herbs is essential. Currently, organic solvents or inorganic acids are commonly used in the conventional method for extracting alkaloids from herbs [9,15]. However, these traditional solvents (such as methanol, ethanol, chloroform, and hydrochloric, nitric, and sulfuric acid) are known for their toxicity, explosiveness, and environmental harm. Therefore, developing eco-friendly and sustainable extraction solvents for natural products is vital to safeguard the environment and consumers while simultaneously boosting the ecological, economic, and innovative competitiveness of industries [16,17].

Currently, most of the research on extracting these protoberberine-type alkaloids applying green solvents employs ionic liquids and deep eutectic solvents (DESs) [16,18,19,20,21]. However, aqueous solutions of carboxylic acids that are inexpensive and safe have not been widely investigated. In our previous research, among the solvents tested, lactic acid, a carboxylic acid, demonstrated the highest extraction efficiency for extracting berberine from *Coscinium fenestratum* [16]. These findings highlight the significant potential of carboxylic acids in extracting alkaloids from medicinal herbs.

In this study, we examined a range of solvents, including volatile organic solvents, inorganic acid solutions, surfactants, DESs, and supramolecular deep eutectic solvents (SUPRADESs), via ultrasonic extraction. More specifically, we explored using aqueous solutions of carboxylic acids to extract protoberberine-type alkaloids and total alkaloids from *C. chinensis*. Significantly, unlike volatile organic solvents and inorganic acids as controls, these green solvents sustain advantageous properties, including economic viability, non-toxicity and biodegradability, environmental compatibility, and heightened efficiency. Remarkably, they are approved for use in pharmaceutical industries, food, and cosmetics [16]. The optimal solvent was identified to enhance extraction efficiency, and the key influencing factors were refined through the response surface method (RSM). Following this, the recovery of alkaloids from the extract solutions was achieved using various macroporous resins. Additionally, both the extraction solvent and the resin underwent a recycling process.

## 2. Results and Discussion

### 2.1. Optimization of Solvent Selection for Extraction of Berberine, Palmatine, and Coptisine

The physicochemical characteristics of extraction solvents—including factors such as polarity, viscosity, solubility, pH, hydrogen bonding capability, and their interactions with target compounds—have been demonstrated to significantly affect the extraction efficiency of bioactive substances from natural products [22]. In this study, aqueous solutions of carboxylic acids, surfactants, DESs, and SUPRADESs were prepared as cost-effective and environmentally friendly alternatives to organic solvents, aiming to identify the most effective extraction solvent.

#### 2.1.1. Effect of Organic Solvents and Inorganic Acids and Bases

**Figure 1:**
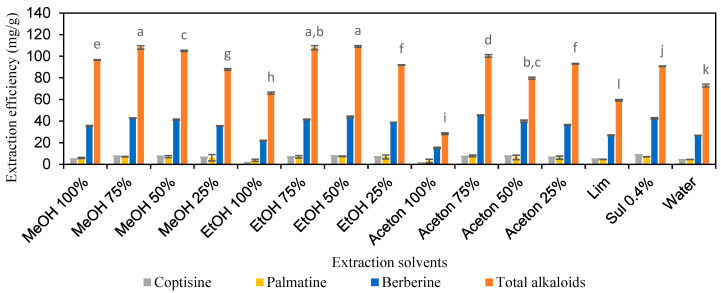
The extraction efficiency of alkaloids from *Coptis chinensis* by solutions of organic solvents and inorganic acids and bases. Different letters above each column indicate statistically significant differences (*p* < 0.05).

To extract alkaloids from *C. chinensis*, volatile organic solvents were prepared at varying concentrations. Methanol (MeOH) was prepared at 99.5%, 75%, 50%, and 25% *w*/*w*, as were ethanol (EtOH) and acetone (Ace), as detailed in Table 1. A limewater solution (Lim) and an aqueous solution of sulfuric acid (Sul) were additionally utilized. These solvents acted as controls. The results are presented in Figure 1 and Table S1, showing extraction efficiencies ranging from 3.0 to 7.8 mg/g for coptisine, from 3.7 to 7.8 mg/g for palmatine, from 15.2 to 45.4 mg/g for berberine, and from 28.3 to 109.1 mg/g for total alkaloids. Markedly, the solvents 75% MeOH, 75% EtOH, and 50% EtOH exhibited the highest extraction efficiencies of total alkaloids, achieving yields of 108.2, 107.9, and 109.1 mg/g, respectively (*p* > 0.05).

#### 2.1.2. Effect of Surfactant Solutions

**Figure 2:**
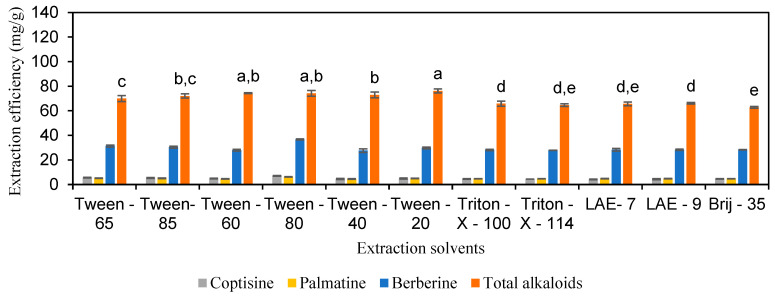
The extraction efficiency of alkaloids from *Coptis chinensis* by solutions of surfactants. Different letters above each column indicate statistically significant differences (*p* < 0.05).

This study investigated the efficiency of surfactants at a concentration of 5 mM in extracting coptisine, palmatine, berberine, and total alkaloids from *C. chinensis*. The results depicted in Figure 2 and Table S2 indicate that the overall extraction efficiency of these alkaloids using surfactants was relatively low. Specifically, the extraction efficiencies for coptisine ranged from 4.2 to 7.1 mg/g, for palmatine from 4.5 to 6.2 mg/g, and for berberine from 27.6 to 36.8 mg/g. The total alkaloid extraction efficiency ranged from 62.8 to 76.2 mg/g. Among the surfactants tested, Tween-60, Tween-80, and Tween-20 showed the highest performance, with total alkaloid extraction efficiencies of 74.3, 74.2, and 76.2 mg/g, respectively, and no statistically significant differences between them (*p* > 0.05).

#### 2.1.3. Effect of Aqueous Solutions of Carboxylic Acids

**Figure 3:**
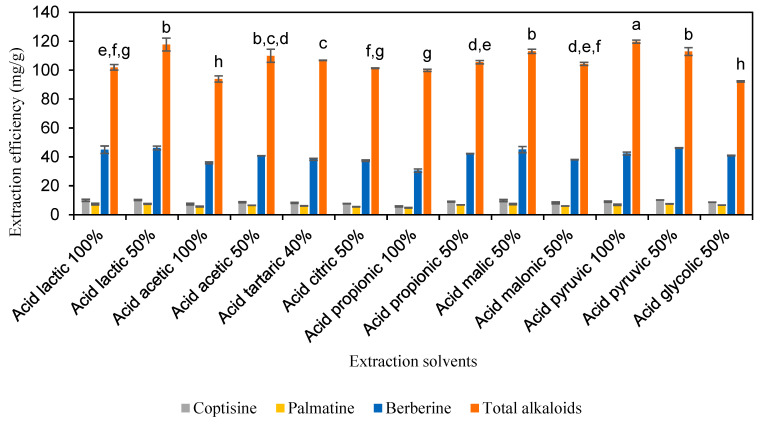
The extraction efficiency of alkaloids from *Coptis chinensis* by solutions of carboxylic acid. Different letters above each column indicate statistically significant differences (*p* < 0.05).

The extraction efficiencies of alkaloids from *C. chinensis* by utilizing thirteen types of carboxylic acid solutions are displayed in Figure 3 and Table S3. As indicated in Figure 3, the extraction efficiencies for acid solutions ranged from 5.7 to 10.2 mg/g for coptisine, from 4.9 to 7.6 mg/g for palmatine, from 30.5 to 46.2 mg/g for berberine, and from 92.2 to 119.8 mg/g for total alkaloids. Significantly, the extraction efficiency of carboxylic acids was higher than that of the organic solvents tested. Among the carboxylic acid solutions, 50% LA, 50% MA, and 98% PA achieved the highest extraction efficiencies for total alkaloids, with values of 111.7, 113.0, and 119.8 mg/g, respectively.

#### 2.1.4. Effect of Aqueous Solutions of DESs and SUPRADESs

**Figure 4:**
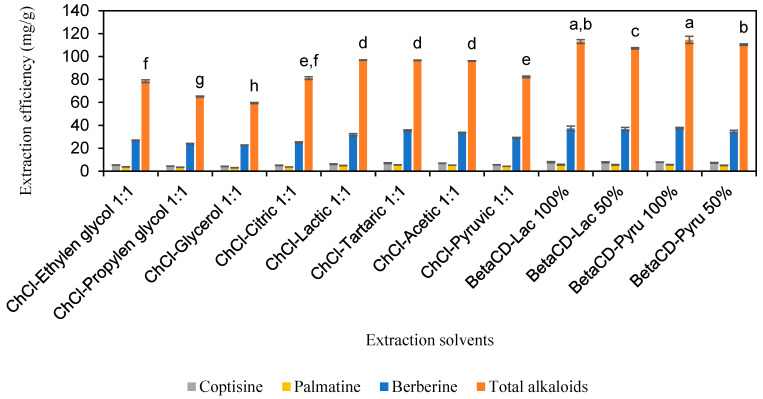
The extraction efficiency of alkaloids from *Coptis chinensis* by solutions of deep eutectic solvents and supramolecular deep eutectic solvents. Different letters above each column indicate statistically significant differences (*p* < 0.05).

DESs and SUPRADESs represent a new generation of solvents emerging within the framework of green chemistry, addressing the limitations of traditional solvents. DESs are composed of two or more components, with one acting as the hydrogen bond donor (HBD) and the other as the hydrogen bond acceptor (HBA), leading to the formation of a eutectic liquid mixture at room temperature. Additionally, SUPRADESs are formulated with cyclodextrins as the HBA in combination with the HBD. These solvents exhibit numerous advantageous properties and offer a promising alternative to conventional organic solvents for extraction processes [16,19,23,24]. Thus, a comparative analysis was conducted to evaluate the efficacy of extracting protoberberine-type alkaloids from *C. chinensis* using aqueous solutions of carboxylic acids and their corresponding DESs and SUPRADESs at various concentrations (*w*/*w*). In addition, DESs formulated from polyalcohols were also investigated. The results are detailed in Figure 4 and Table S4. The results show that the extraction efficiencies for coptisine ranged from 4.2 to 8.0 mg/g, for palmatine from 3.1 to 5.7 mg/g, for berberine from 22.7 to 37.5 mg/g, and for total alkaloids from 59.4 to 114.5 mg/g. The highest total alkaloid extraction efficiencies were observed in the 100% β-CD-PA and 100% β-CD-LA systems, at 114.5 mg/g and 107.1 mg/g, respectively, with no statistically significant difference between them (*p* > 0.05). It was observed that DESs containing polyalcohols as the HBD component were less efficient compared to those with carboxylic acids as the HBD component. Moreover, SUPRADESs generally demonstrated higher extraction efficiencies than DESs. The extraction performance using SUPRADESs surpassed that of organic solvents and inorganic acids and bases. However, SUPRADESs did not enhance extraction efficiency beyond that achieved with solvents containing only individual carboxylic acids. Thus, the carboxylic acid component in the SUPRADESs primarily influences the efficiency of alkaloid extraction from *C. chinensis*. Solvents containing only carboxylic acid, rather than the two-component systems in SUPRADESs, would be more cost-effective and facilitate recovery.

Based on the extraction results using surfactant solutions, carboxylic acids, DESs, SUPRADESs, and control solvents (organic solvents and inorganic acids and bases), carboxylic acid solutions showed the highest extraction efficiency for coptisine, palmatine, berberine, and total alkaloids from *C. chinensis*. Specifically, 50% lactic acid, 50% malic acid, and 98% pyruvic acid provided the highest total alkaloid extraction efficiencies, achieving 117.8 ± 4.5 mg/g, 113.0 ± 1.4 mg/g, and 119.8 ± 1.0 mg/g, respectively, with no statistically significant differences (*p* > 0.05). Thus, they were chosen as the solvents for the further optimization of influencing conditions.

Coptisine, palmatine, and berberine are weakly basic alkaloids. Using aqueous solutions of organic acids to extract alkaloids from *C. chinensis* is well aligned with the acid–base extraction principle. Acids can react with alkaloids in their free base form and convert them into alkaloid salts, thereby improving their solubility in the extraction solvent and enhancing efficiency. Therefore, the presence of acid molecules improves the interaction between the solvent and solute, which explains the observed high efficiency and selectivity [25]. Specifically, acids such as lactic acid, malic acid, and pyruvic acid exhibiting the highest efficiency of alkaloid extraction from *C. chinensis* can be attributed to the formation of lactate, malate, and pyruvate salts of the alkaloids. These salts benefit from increased polarity in their respective acidic solutions, which increases solubility and enhances alkaloid extraction efficiency.

### 2.2. Design of Optimal Conditions for Extraction of Coptisine, Palmatine, Berberine, and Total Alkaloids from Coptis Chinensis

Following the identification of lactic acid, malic acid, and pyruvic acid as the optimal extraction solvents, this study systematically investigated the individual factors influencing the extraction efficiency of coptisine, palmatine, berberine, and total alkaloids from *C. chinensis*. The critical parameters examined included the concentration of lactic acid, malic acid, and pyruvic acid (% *w*/*w*), the liquid-to-solid ratio (mL/g), the extraction time (min), and the extraction temperature. Each factor was assessed for its impact on the efficiency of alkaloid extraction, with the aim of optimizing the extraction process for the maximum yield of the target alkaloids. Initially, a thorough single-factor investigation was performed to evaluate the effect of each variable on extraction yields. Based on the findings from this analysis, the RSM was subsequently utilized to optimize the key conditions affecting extraction efficiency and overall performance. This approach has been extensively employed in various studies, particularly in extraction, due to its effectiveness in reducing the time required to identify optimal processing conditions [19].

#### 2.2.1. Effect of Carboxylic Acid Concentration

Optimizing the concentration of carboxylic acids is crucial for enhancing the extraction efficiency of alkaloids. In this study, various concentrations were evaluated: 0%, 20%, 40%, 60%, 80%, and 96% *w*/*w* for lactic acid, 0%, 20%, 40%, 60%, 80%, and 98% for pyruvic acid, and 0%, 10%, 20%, 30%, 40%, and 50% *w*/*w* for malic acid (solid form at room temperature). The extraction conditions were kept constant with a liquid-to-solid ratio of 20 mL/g, an extraction time of 30 min, and an extraction temperature of 50 °C.

**Figure 5:**
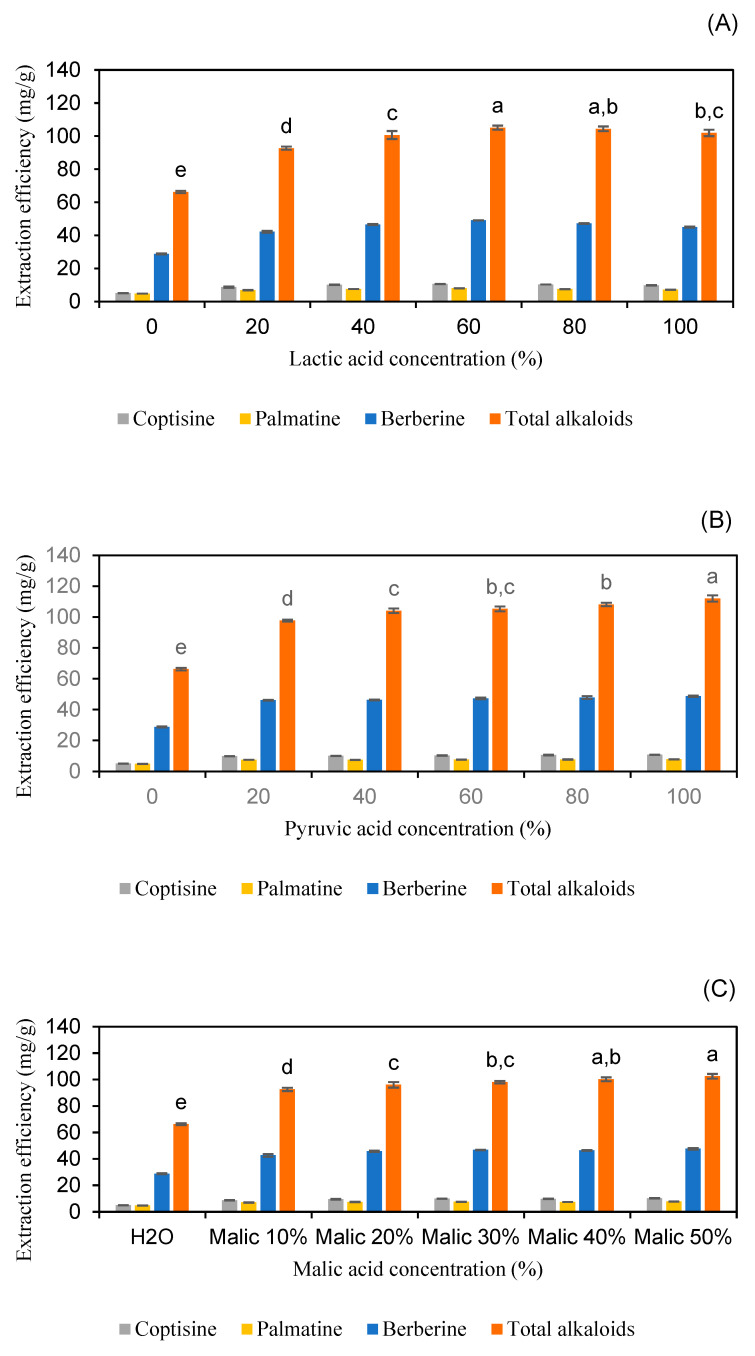
The extraction efficiency of alkaloids from *Coptis chinensis* using varying concentrations of lactic acid (**A**), pyruvic acid (**B**), and malic acid (**C**). Different letters above each column indicate statistically significant differences (*p* < 0.05).

The results indicated that the extraction efficiencies of coptisine, palmatine, berberine, and total alkaloids were highest at 60% and 80% (*w*/*w*) for lactic acid, with no significant difference between these concentrations (*p* > 0.05). For malic acid, the extraction efficiency peaked at concentrations of 40% and 50% (*w*/*w*), with no statistically significant difference between these values (*p* > 0.05). In the case of pyruvic acid, the highest extraction efficiencies were observed at 80% and 98% (*w*/*w*), with no statistically significant difference between these concentrations (*p* > 0.05) (Figure 5 and Table S5). Thus, considering economic factors, 60% lactic acid, 40% malic acid, and 80% pyruvic acid aqueous solutions were selected for further optimization studies.

These findings are consistent with the literature. Jiang et al. reported that the extraction efficiency of different types of bioactive alkaloids was significantly influenced by the concentration of deep eutectic solvents, with optimal yields observed at specific solvent concentrations [21]. Similarly, Dai et al. demonstrated that natural deep eutectic solvents at certain concentrations enhanced the extraction of bioactive compounds, highlighting the importance of solvent concentration in extraction processes [26]. Furthermore, extraction efficiency is influenced by the plant matrix, as interactions between the solvent and the plant material can affect the release of target compounds. This underscores the need to optimize solvent concentration based on the specific characteristics of the solvent and the plant material [27].

#### 2.2.2. Effect of Liquid–Solid Ratio

**Figure 6:**
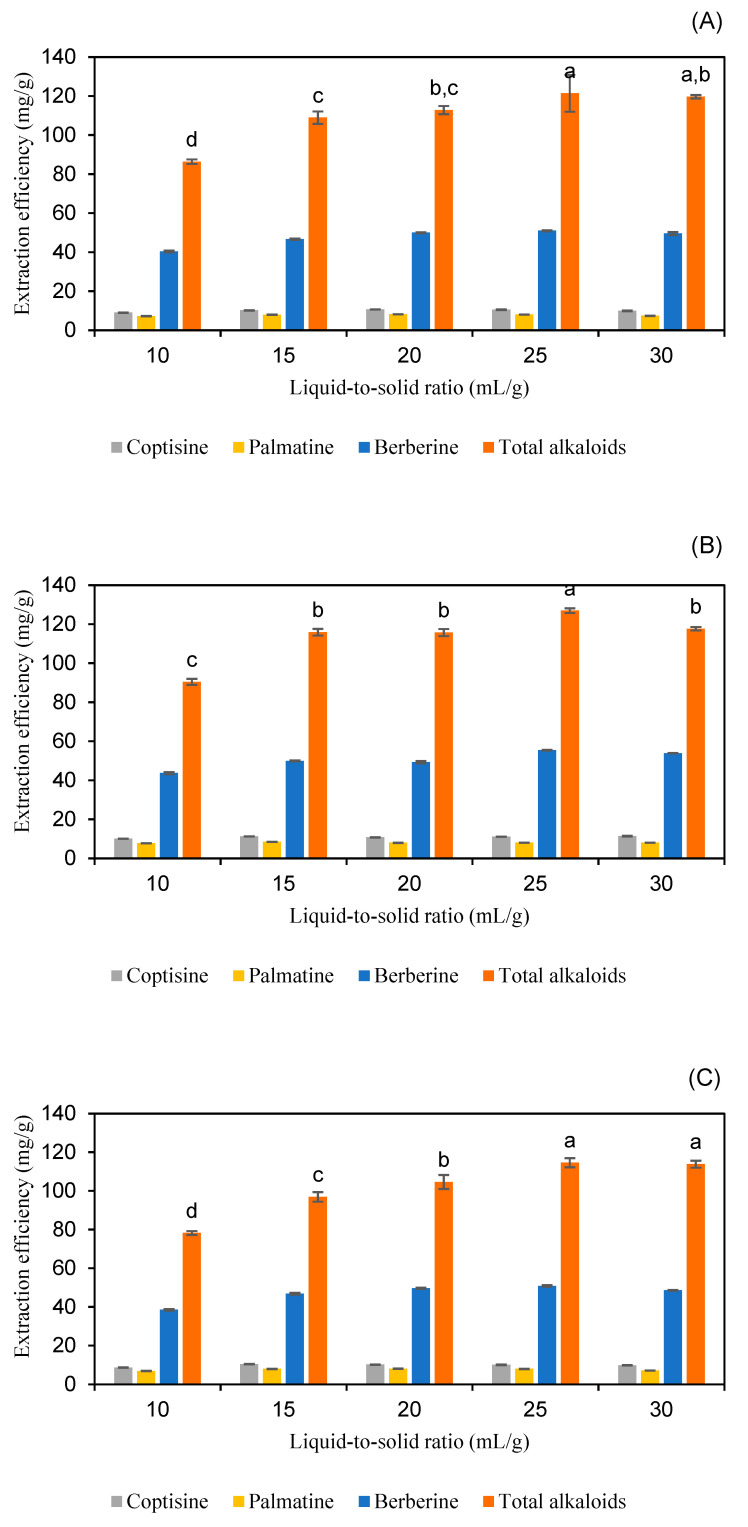
The extraction efficiency of alkaloids from *Coptis chinensis* using varying liquid-to-solid ratios of lactic acid (**A**), pyruvic acid (**B**), and malic acid (**C**) for each sample. Different letters above each column indicate statistically significant differences (*p* < 0.05).

Regarding the extraction process, traditional liquid extraction methods are often inefficient due to the requirement for low solid–liquid ratios and prolonged extraction durations. The use of low solid–liquid ratios can lead to increased solvent consumption, which contradicts the principles of green chemistry, whereas an inadequate liquid-to-solid ratio may hinder the complete extraction of desired substances from medicinal herbs. Consequently, extraction efficiency can be enhanced by optimizing solid–liquid ratios [16,28,29]. In this experiment, liquid-to-solid ratios of 10, 15, 20, 25, and 30 mL/g were tested while maintaining constant extraction conditions: a 30 min extraction time, an extraction temperature of 50 °C, and solvents comprising either 60% *w*/*w* lactic acid, 40% *w*/*w* malic acid, or 80% *w*/*w* pyruvic acid solutions. The results, presented in Figure 6 and Table S6, show that the highest total alkaloid extraction efficiency using 60% *w*/*w* lactic acid was achieved at a liquid-to-solid ratio of 25 mL/g, with this result being statistically significant (*p* < 0.05). For 40% *w*/*w* malic acid, the maximum extraction efficiency for total alkaloids was observed at liquid-to-solid ratios of 25 mL/g and 30 mL/g, with no statistically significant difference between these values (*p* > 0.05). Similarly, for 80% *w*/*w* pyruvic acid, the highest extraction efficiency occurred at a liquid-to-solid ratio of 25 mL/g, significantly different from other ratios (*p* < 0.05). Consequently, a liquid-to-solid ratio of 25 mL/g was selected for all solvents in subsequent investigations.

Adjusting the liquid-to-solid ratio is important to balance solvent usage and extraction efficiency. According to Xia et al., increasing the liquid-to-solid ratio from 5:1 to 20:1 resulted in an approximately 50% increase in the oxymatrine extraction yield from *Sophora flavescens*, indicating the critical role of the liquid-to-solid ratio in extraction efficiency [30]. Similarly, a study on the extraction of solasodine from *Solanum* species found that the extraction efficiency was enhanced with increasing liquid-to-solid ratios, reaching a peak at a 42:1 ratio [31]. However, it is important to note that excessively high liquid-to-solid ratios may not lead to proportional increases in extraction yields and can result in unnecessary solvent usage, contradicting green chemistry principles. Therefore, selecting an optimal liquid-to-solid ratio is essential to maximize extraction efficiency while minimizing solvent consumption.

#### 2.2.3. Effect of Extraction Time

**Figure 7:**
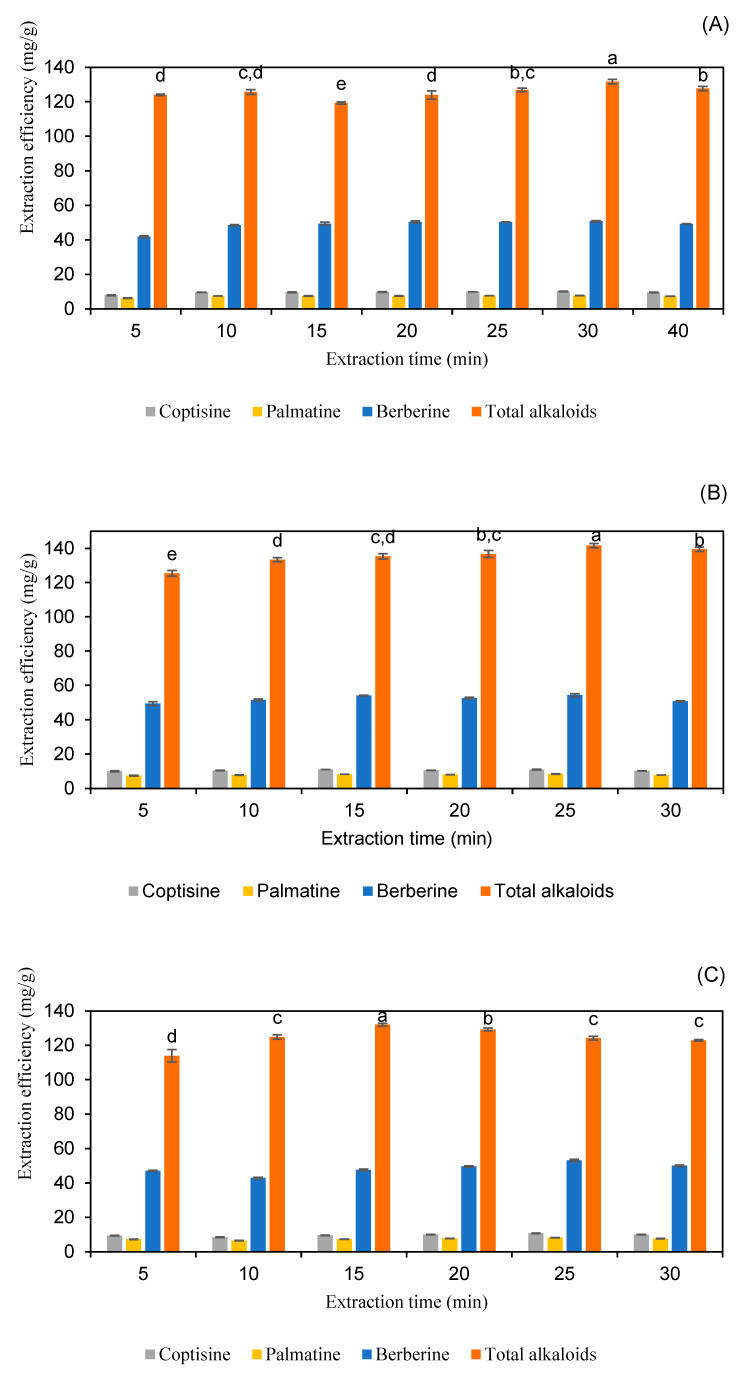
The extraction efficiency of alkaloids from *Coptis chinensis* using different extraction times with lactic acid (**A**), pyruvic acid (**B**), and malic acid (**C**). Different letters above each column indicate statistically significant differences (*p* < 0.05).

Finding the ideal extraction time is essential to achieving maximum efficiency while preventing compound degradation and minimizing operational expenses. If the extraction period is too short, the interaction between the solvent and the sample may be insufficient, leading to incomplete alkaloid solubilization and reduced yield. On the other hand, excessive extraction time can cause bioactive compounds to degrade due to prolonged heat exposure or hydrolysis. Additionally, extended processing increases energy consumption and overall costs, making it less viable for large-scale industrial applications [32]. The impact of extraction time on alkaloid yield was assessed at intervals of 5, 10, 15, 20, 25, 30, and 40 min, with other parameters held constant. As detailed in Figure 7 and Table S7, the extraction efficiency reached its maximum at 30 min using 60% *w*/*w* lactic acid, at 15 min using 40% *w*/*w* malic acid, and at 25 min using 80% *w*/*w* pyruvic acid, all with a liquid-to-solid ratio of 25 mL/g. These results indicate that the optimal extraction time varies depending on the solvent system used, likely due to differences in solvent polarity and the strength of interactions between alkaloids and the sample matrix.

These results align with previous research findings. Li et al. (2021) observed that berberine extraction from *C. chinensis* was most effective within the 20–30 min range, beyond which degradation became evident [20]. Based on the study by Kopp et al. on the extraction and analysis of pyrrolizidine alkaloids in medicinal plants, extraction time significantly impacts both yield and compound stability. The study found that extending extraction time initially increased alkaloid recovery, but after reaching an optimal duration, further prolongation did not enhance yields significantly. Instead, prolonged extraction times increased the risk of the oxidation and structural degradation of alkaloids, leading to a decline in their bioactive properties. These findings highlight the importance of optimizing extraction time to balance yield maximization and compound preservation. Selecting an appropriate extraction time is crucial to preventing unnecessary degradation while ensuring efficient alkaloid recovery [33].

#### 2.2.4. Effect of Extraction Temperature

**Figure 8:**
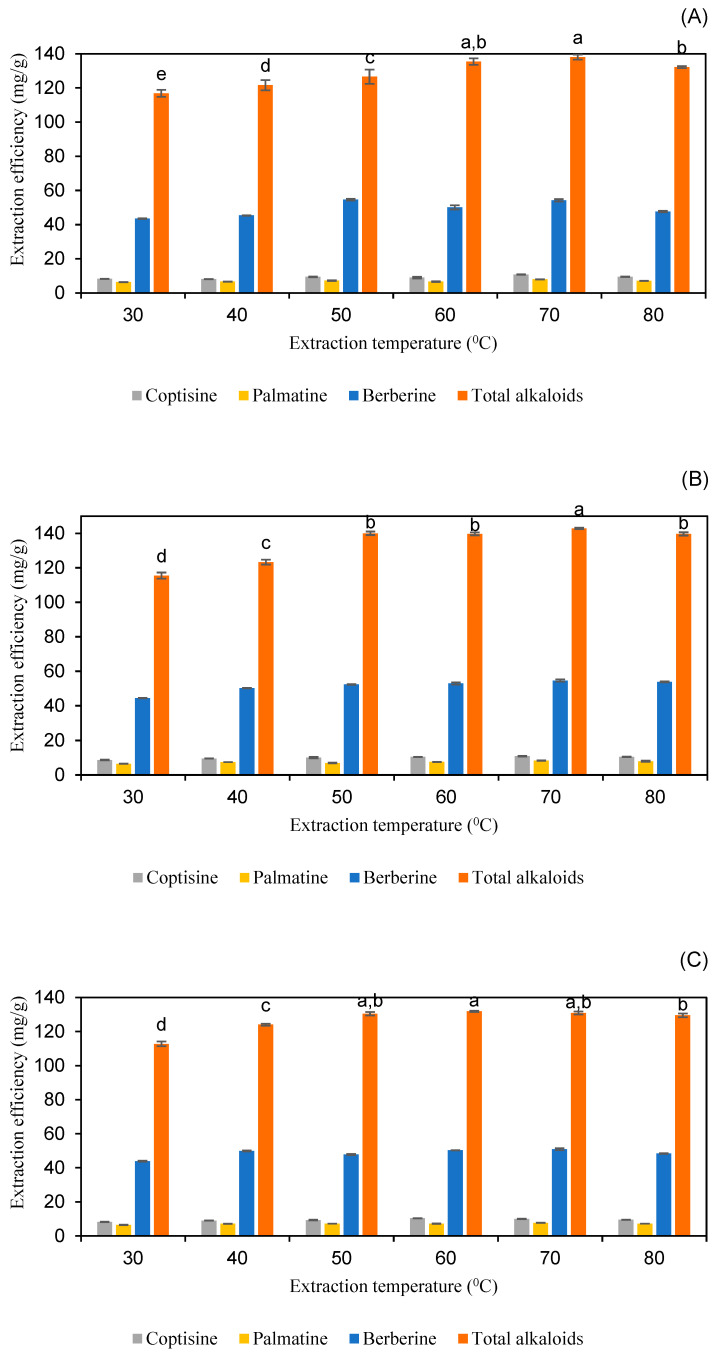
The extraction efficiency of alkaloids from *Coptis chinensis* using different extraction temperatures with lactic acid (**A**), pyruvic acid (**B**), and malic acid (**C**). Different letters above each column indicate statistically significant differences (*p* < 0.05).

Extraction temperature is a critical factor in determining extraction efficiency. Higher temperatures generally enhance solvent power by reducing viscosity and increasing diffusivity. The lowering of surface tension and weakening of interactions between the target compounds and the sample matrix due to elevated temperatures can improve the desorption and dissolution of the target compounds into the solvent. However, the thermal degradation of compounds can occur at excessively high temperatures [34,35]. Extraction temperatures of 30, 40, 50, 60, 70, and 80 °C were evaluated while maintaining constant extraction conditions. As shown in Figure 8 and Table S8, employing 60% *w*/*w* lactic acid for 30 min or 40% *w*/*w* malic acid for 15 min resulted in the highest extraction efficiencies for coptisine, palmatine, berberine, and total alkaloids at temperatures of 60 °C and 70 °C, with no statistically significant difference between these temperatures. For 80% *w*/*w* pyruvic acid, extraction efficiency peaked at 70 °C when the duration of extraction was 25 min. These results align with previous studies on alkaloid extraction, where moderate temperatures (50–70 °C) were found to be optimal for maximizing yield while minimizing thermal degradation. For instance, Li et al. [20] reported that berberine extraction from *C. chinensis* was most efficient at 60 °C, with significant degradation observed at temperatures above 70 °C. Similarly, Qiuhong et al. investigated the influence of extraction temperature on alkaloid yield across a temperature range of 40 °C to 100 °C. The results demonstrated that higher temperatures initially facilitated improved extraction efficiency. However, when the temperature exceeded 60 °C, a decline in alkaloid yield was observed, potentially due to thermal degradation. Based on these findings, 60 °C was identified as the optimal extraction temperature to balance efficiency and compound stability [36]. Considering both economic practicality and the preservation of bioactive components [16], 60 °C was selected as the optimal extraction temperature for lactic acid and malic acid, while 70 °C was chosen for pyruvic acid.

A single-factor survey was conducted to identify influencing factors as a precursor to optimizing extraction conditions. To better understand the impact of various factors and their interactions on the extraction process, further research was conducted using the RSM. It was observed that the time factor did not significantly affect the efficiency of alkaloid extraction from *C. chinensis*, as the differences in efficiency across different time points were negligible. Therefore, subsequent optimization focused on the factors of acid concentration (A), the liquid-to-solid ratio (B), and extraction temperature (D), using the Box–Behnken model to determine the most optimal extraction conditions.

#### 2.2.5. Optimization of Extraction Conditions Using Response Surface Methodology

To elucidate the influence of various factors on the extraction process and their interactions, the RSM was employed to optimize the conditions affecting extraction performance. The Box–Behnken experimental model was used to optimize the total alkaloid extraction process in *C. chinensis*, designed using Design Expert 13 software. The independent variables investigated included acid concentration (A), the liquid-to-solid ratio (B), and extraction temperature (D), as presented in Table 2. The model consisted of 17 experimental units, including 5 central experiments.

##### Lactic Acid

Experiments were conducted to extract total alkaloids from *C. chinensis* under 17 designed experimental conditions using lactic acid as the solvent. The analysis of reliability and variance results is shown in Table S9. The Fisher F-test model value of 44.18 and probability value (*p*) < 0.0001 indicated that the experimental model was established with high statistical significance. The correlation coefficient (R^2^) value of 0.9827 and the model’s lack-of-fit value of 0.2681 (>0.05) demonstrated the compatibility between the model and the experiment. The final regression equation represented the relationship between total alkaloid extraction efficiency and the variables of the Box–Behnken response surface quadratic model, as described in Equation (1), with a high coefficient of determination (R^2^ = 0.9827).

Y = 3.93845 + 0.009498A + 0.405847B + 0.0007277D + 0.001037AB − 8.79172 × 10^−6^AD − 0.000099BD − 0.000015A2 − 0.007270B2 − 9.67325 × 10^−6^D2 (1)

where Y represents total alkaloid extraction efficiency (%), A is lactic acid concentration (%), B is the liquid-to-solid ratio (mL/g), and D is extraction temperature (°C). Equation (1) shows high compatibility between experimental and predicted values, with extraction efficiencies obtained from experiments and calculated using Equation (1) exhibiting a high correlation (R^2^ = 0.9827). The regression coefficients indicating the influence of independent variables on extraction efficiency are presented in Table S9. The linear factors A and B’s values, interaction pair AB, and quadratic value B2 all demonstrated high significance within the model, significantly affecting extraction efficiency (*p* < 0.05).

**Figure 9:**
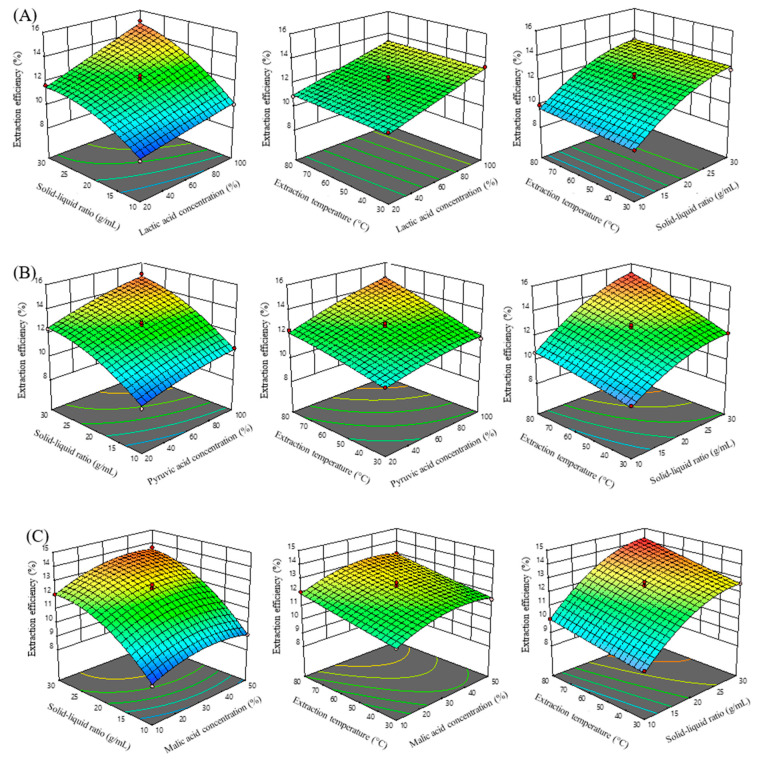
Response surface plots of total alkaloid extraction, illustrating the optimization of extraction conditions using lactic acid (**A**), pyruvic acid (**B**), and malic acid (**C**) as solvents.

The response surface plot illustrates the interaction between the independent variables as well as between the independent and dependent variables. Figure 9A demonstrates the influence of various factors on the extraction efficiency of total alkaloids from *C chinensis*. It can be observed that except for the interaction surface between temperature and extraction concentration, the response surfaces of the other factors have significant slopes, indicating that these factors greatly impact the extraction process, consistent with the variance analysis results in Table S9.

The optimal extraction conditions, derived from response surface model optimization, are as follows: a lactic acid concentration of 100%, liquid-to-solid ratio of 30 mL/g, and extraction temperature of 60.89 °C. Under these conditions, the expected total alkaloid extraction efficiency is 136.6 mg/g. To align with laboratory equipment constraints, the actual survey values were adjusted to a lactic acid concentration of 96%, a liquid-to-solid ratio of 30 mL/g, and an extraction temperature of 60 °C. Verification experiments under these optimal conditions yielded a total alkaloid extraction efficiency of 139.6 mg/g, with no statistically significant difference between experimental and predicted results (*p* > 0.05).

##### Pyruvic Acid

The efficiencies of the extraction of total alkaloids from *C. chinensis* utilizing pyruvic acid as the solvent were evaluated under 17 different experimental conditions.

Table S10 presents the reliability and variance analysis results. The Fisher F-test yielded a model value of 74.11 with a probability value (*p*) < 0.0001, indicating a highly statistically significant experimental model. The correlation coefficient (R^2^) of 0.9896 and lack-of-fit value of 0.4021 (>0.05) suggested strong compatibility between the model and the experimental data. The final regression equation, described in Equation (2), captures the relationship between total alkaloid extraction efficiency and the variables of the Box–Behnken response surface quadratic model, with a high coefficient of determination (R^2^ = 0.9896).

Y = 4.35739 + 0.011896A + 0.315225B + 0.010585D + 0.000454AB + 0.000304AD + 0.001597BD − 0.000129A2 − 0.006440B2 − 0.000188D2 (2)

where Y represents total alkaloid extraction efficiency (%), A is pyruvic acid concentration (%), B is the liquid-to-solid ratio (mL/g), and D is the extraction temperature (°C).

Equation (2) demonstrates the alignment between the experimental and predicted values, with the extraction efficiency obtained from experiments showing a high correlation coefficient of determination (R^2^ = 0.9896), as depicted in Figure 9B and Table S10, revealing the regression coefficients and the influence of independent variables on extraction efficiency. The values of the linear factors (A, B, D), interaction pairs (AD, BD), and the quadratic term (B^2^) all displayed high reliability within the model, considerably impacting extraction efficiency (*p* < 0.05). The interactions between independent variables and their effect on the dependent variable are illustrated by the response surface plot. Figure 9B shows the impact of various factors on the efficiency of total alkaloid extraction from *C. chinensis* using pyruvic acid. Except for the interaction surface between temperature and extraction concentration, the response surfaces for the remaining factors display pronounced slopes, indicating a substantial influence on the extraction process. This observation aligns with the variance analysis results presented in Table S10. From the optimization process using the response surface model, the optimal extraction conditions were identified as a pyruvic acid concentration of 87.51%, a solvent-to-drug ratio of 29.30 mL/g, and an extraction temperature of 73.99 °C. Under these conditions, the predicted total alkaloid extraction yield was 144.6 mg/g. To match laboratory equipment constraints, these values were adjusted to a pyruvic acid concentration of 88%, a solvent-to-drug ratio of 30 mL/g, and an extraction temperature of 75 °C. Verification experiments under these adjusted conditions achieved a total alkaloid extraction efficiency of 146.3 ± 0.4 mg/g, showing no statistically significant difference from the predicted results (*p* > 0.05).

##### Malic Acid

Trials to extract total alkaloids from *C. chinensis* were carried out under 17 designed experimental conditions, employing malic acid as the solvent. Reliability and variance analysis results are presented in Table S11. The Fisher F-test model value of 111.71 and probability value (*p*) < 0.0001 confirmed the high statistical significance of the experimental model. The correlation coefficient (R^2^) value of 0.9931 and the model’s lack-of-fit value of 0.5972 (>0.05) showed the compatibility between the model and the experiment. The final regression equation representing the relationship between total alkaloid extraction efficiency and Box–Behnken response surface quadratic model variables is described in Equation (3), with a high coefficient of determination (R^2^ = 0.9931).

Y = 1.91458 + 0.093360A + 0.490037B + 0.010737D + 0.000888AB + 0.000027AD +0.000455BD − 0.001463A2 − 0.008957B2 + 0.000041D2 (3)

where Y is the total alkaloid extraction efficiency (%), A is the malic acid concentration (%), B is the liquid-to-solid ratio (mL/g), and D is the extraction temperature (°C). This equation exhibits strong agreement between the experimental and predicted values, as indicated by the extraction efficiency obtained from experiments and calculated using Equation (3), which showed a high correlation coefficient of determination (R^2^ = 0.9931) (Figure 9C).

The regression coefficients pointing to the impact of independent variables on extraction efficiency were calculated and are displayed in Table S11. The linear factors (A, B, D) and the quadratic terms (A^2^, B^2^) exhibited high reliability within the model and had a significant effect on extraction efficiency (*p* < 0.05).

The response surface plot illustrates the interactions among the independent variables, as well as between the independent and dependent variables. The impact of various factors on the efficiency of total alkaloid extraction is depicted in Figure 9C.

Except for the interaction surface between temperature and extraction concentration, the response surfaces of other factors all have a large slope, indicating that these factors have a significant impact on the extraction process, consistent with the results of variance analysis in Table S11.

Based on the optimization process data obtained using the response surface model, the optimal extraction conditions are as follows: a malic acid concentration of 40.04%, liquid-to-solid ratio of 29.90 mL/g, and extraction temperature of 80.00 °C. The expected total alkaloid extraction yield under these conditions is 133.1 mg/g. To accommodate laboratory equipment conditions, the actual survey values were adjusted to a malic acid concentration of 40%, a liquid-to-solid ratio of 30 mL/g, and an extraction temperature of 80 °C. In the verification experiments conducted under optimal conditions, the total alkaloid extraction efficiency was determined to be 133.0 ± 0.5 mg/g. Statistical analysis revealed no significant difference between the experimental outcomes and the predicted values (*p* > 0.05).

Collectively, compared to the best outcomes of extraction efficiency using organic solvents under identical optimal conditions, these results demonstrate a high level of competitiveness. They highlight the potential of using aqueous carboxylic acid solutions, in conjunction with ultrasound support, as a viable and eco-friendly alternative for extracting alkaloids from therapeutic botanicals. This approach presents a promising substitute for the traditional use of organic solvents and inorganic acid–base solutions.

The literature on alkaloid extraction from *C. chinensis* reports a variety of methodologies. Regarding conventional extraction, the utilization of an ethanol concentration of 45%, extraction time of 133 min, and liquid-to-solid ratio of 42 *v*/*w* yielded a total alkaloid content of 159.6 ± 0.15 mg berberine chloride equivalent/g (BCE/g) [37]. Hui Teng and Yong Hee Choi utilized an ethanol concentration of 59%, an extraction time of 47 min, and a temperature of 66.22 °C, resulting in reported total alkaloid, berberine, and palmatine yields of 165.7 mg BCE/g, 76.9 mg BCE/g, and 14.6 mg BCE/g, respectively [38]. Under the optimal conditions of a 0.34% phosphoric acid concentration, an extraction time of 300 min, and a liquid-to-solid ratio of 51.45 mL/g, the obtained total alkaloid, berberine, and palmatine yields were 207.10 mg BCE/g, 72.48 mg BCE/g, and 15.77 mg palmatine chloride equivalent/g, respectively [39]. Mostly, conventional extraction methods consume large amounts of organic solvents and require extended processing times.

Regarding green extraction methods, Lijing Li et al. utilized DESs based on choline chloride and phenol with a molar ratio of 1:3, a water content of 30%, and a liquid-to-solid ratio of 30 g/mL. Extraction was performed for 30 min at 60 °C to extract palmatine and berberine from *C. chinensis*, resulting in yields of 16.71 mg/g and 57.40 mg/g, respectively [20]. Yuanbin Li et al. extracted alkaloids from *Coptidis rhizoma* using a 0.6 M betaine–tartaric acid–water DES, with a liquid-to-solid ratio of 35:1 and an extraction time of 29.5 min, resulting in an alkaloid concentration of 128.43 mg/g [19]. In our study, the use of aqueous solutions of carboxylic acids to extract alkaloids under optimal conditions resulted in higher total alkaloid extraction efficiencies than DESs and other solvents, with a value of 146.3 mg/g. In fact, research on the extraction of protoberberine-type alkaloids from medicinal plants using green organic acids is limited. To the best of our knowledge, this is the first effort to comprehensively study the effectiveness of carboxylic acids in extracting protoberberine-type alkaloids from *C. chinensis*.

### 2.3. Recovery of Total Alkaloids from C. chinensis Extract Using Adsorption Resin

**Figure 10:**
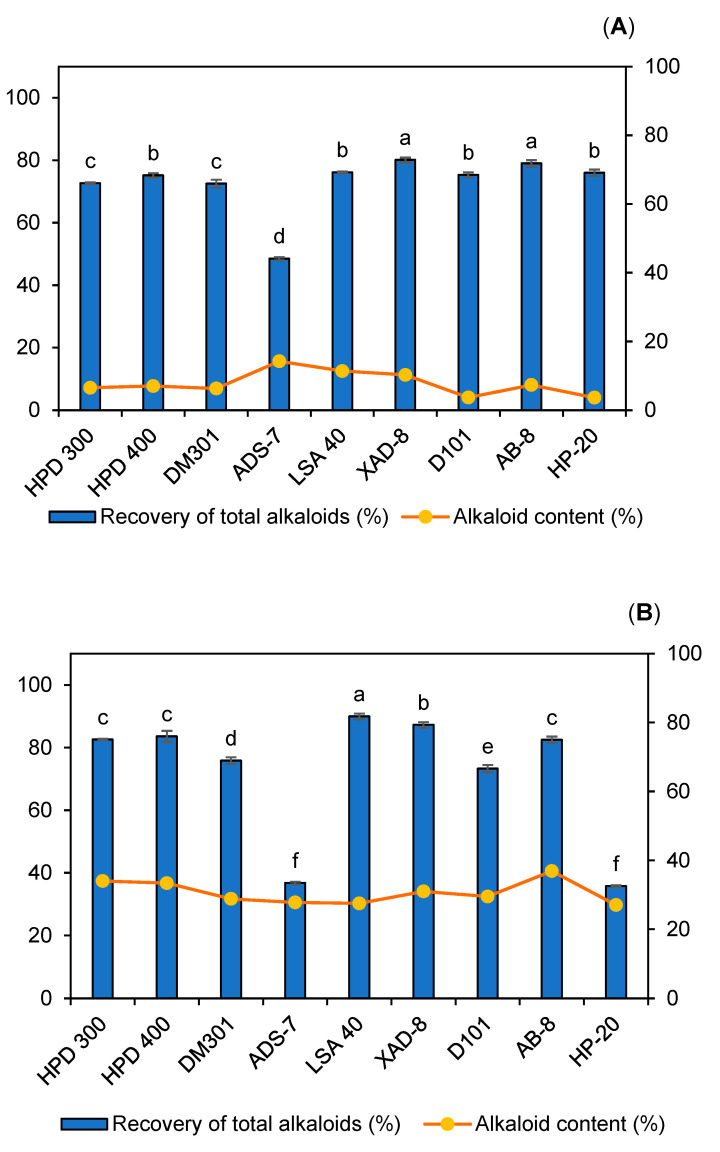

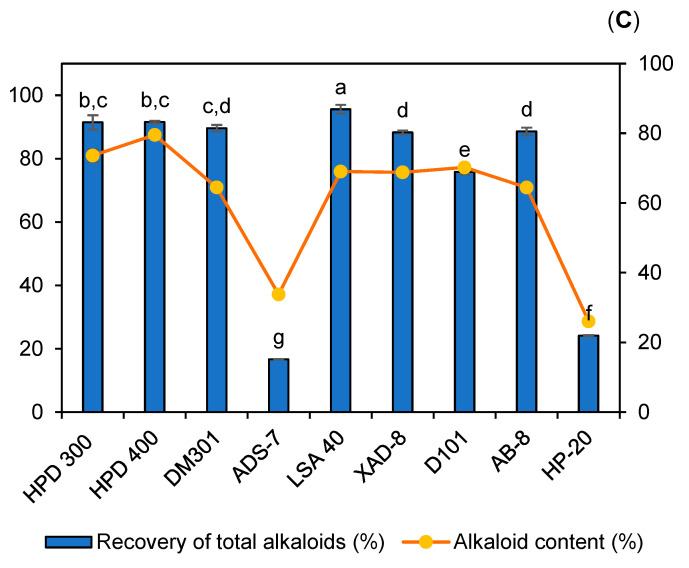
The adsorption and desorption capacities, along with the alkaloid content (%), for the recovery of total alkaloids from the lactic acid extract (**A**), pyruvic acid extract (**B**), and malic acid extract (**C**) of *Coptis chinensis*. Recovery of total alkaloids (%) refers to the percentage of alkaloids removed from the extract through adsorption onto the resin. Alkaloid content (%) refers to the percentage of alkaloids recovered after desorption from the resin using ethanol. Significant differences within the same graph type are indicated by different letters, as determined by the LSD test (*p* < 0.05).

The adsorption capacity of macroporous resins may depend on the physicochemical characteristics of the target compounds as well as the structural features of the resin, including particle size, surface area, pore diameter, and polarity [40]. Selecting a resin requires matching its polarity to that of the target compound, following the principle of “like dissolves like” [41]. Nine different types of macroporous resin were used to recover alkaloids from *C. chinensis* extract, including HPD-300, HPD-400, DM-301, ADS-7, LSA-40, XAD-8, D-101, AB-8, and HP-20. The results, shown in Figure 10, indicate that for the lactic acid solution, resins XAD-8 and AB-8 demonstrated the best recovery efficiency, with recovery rates of 80.11 ± 0.78% and 79.00 ± 1.06%, respectively, with no statistically significant difference between these values (*p* > 0.05). For malic acid and pyruvic acid solutions, the resin LSA-40 showed the most impressive recovery rates, with recovery rates of 95.58 ± 1.40% and 89.86 ± 0.90%, respectively. On the contrary, the ADS-7 resin exhibited the lowest recovery efficiency for total alkaloids across the lactic acid, pyruvic acid, and malic acid solutions.

The alkaloid recovery yield for lactic acid extracts was relatively low, ranging from 3.65% to 14.4%. In contrast, the pyruvic acid extracts demonstrated moderate alkaloid recovery yields, ranging from 27.02% to 36.88%. Notably, malic acid extracts exhibited impressive alkaloid recovery rates. Except for the ADS-7 and HP-20 resins, the remaining resins achieved alkaloid recovery yields ranging from 64.44% to 79.52%. Among these, the HPD-400 resin exhibited the highest selective alkaloid recovery efficiency, yielding 79.52%. The HPD-400 resin demonstrated the highest efficiency of total alkaloid recovery from the malic acid extract of *C. chinensis* compared to the other resins tested, including HPD-300, DM-301, ADS-7, LSA-40, XAD-8, D-101, AB-8, and HP-20. The superior performance of HPD-400 can be attributed to its unique physicochemical properties, including a high surface area, optimal pore size distribution, and moderate polarity. These characteristics make it particularly effective for adsorbing alkaloids, which are relatively small, polar to moderately non-polar molecules. The primary components of the total alkaloids, namely, berberine, palmatine, and coptisine, share structural features such as a quaternary ammonium group and aromatic rings, which contribute to their polarity and affinity for the HPD-400 resin. The resin’s pore size is well suited for accommodating these alkaloid molecules, ensuring efficient adsorption. Additionally, the resin’s balanced hydrophobic–hydrophilic interactions facilitate the selective adsorption of the alkaloids over other co-extracted compounds, resulting in high recovery rates [42]. The combination of these features underscores the HPD-400 resin’s exceptional ability to recover alkaloids from *C. chinensis* extracts with malic acid as the solvent, offering a promising approach for sustainable and efficient alkaloid recovery. This marks the first reported evidence for this approach. Based on these findings, we recommend using malic acid as the extraction solvent in combination with the HPD-400 resin to achieve high alkaloid recovery yields and superior purity for *C. chinensis*.

## 3. Materials and Methods

### 3.1. Plant Materials

Rhizomes of *C. chinensis* Franch. were obtained from a traditional medicine supplier based in Hue, Vietnam. The plant material was identified by Dr. Vu Tien Chinh and Dr. Le Tuan Anh from the Vietnam National Museum of Nature. The specimen, labeled with the code CCF-01, was deposited at the Faculty of Pharmacy, University of Medicine and Pharmacy, Hue University, Vietnam. The dried samples were ground into powders, then sieved through a 0.5–1.0 mm mesh, and stored in a light-protected environment for subsequent extraction experiments.

### 3.2. Chemicals and Reagents

The berberine chloride, palmatine chloride, and coptisine standards were purchased from Sigma-Aldrich Co. (St. Louis, MO, USA) and Macklin Inc. (Shanghai, China).

Citric acid, lactic acid, acetic acid, tartaric acid, succinic acid, glycolic acid, malonic acid, propionic acid, malic acid, and pyruvic acid were provided by Macklin Inc. (Shanghai, China). Choline chloride was procured from Thermo Fisher Scientific, Co. (Waltham, MA, USA). HPD-300, HPD-400, DM-301, ADS-7, LSA-40, XAD-8, D-101, and AB-8 were supplied by Tianjin Haoju Resin Technology Co., Ltd. (Tianjin, China). The HP-20 resin was procured from Supelco™ Analytical (Bellefonte, PA, USA).

The preparation of solvents is detailed in Table 1. The DESs were prepared by directly mixing choline chloride with acids. For the SUPRADESs, cyclodextrin was combined with carboxylic acid. These mixtures were formed with a defined molar ratio and heated below 100 °C for 2–4 h, with continuous stirring to obtain stable, liquid homogeneity. The DESs and SUPRADESs were then allowed to cool at room temperature for 24 h. The solvents were stored in sealed vials and kept in a desiccator.

### 3.3. Quantification of Berberine, Palmatine, Coptisine, and Total Alkaloids

The berberine, palmatine, and coptisine were quantified in all samples using the RP-HPLC instrument (Agilent Technologies, Santa Clara, CA, USA). The HPLC method, described in more detail in the literature, was adopted with slight modifications [19]. The HPLC was equipped with an Agilent Eclipse XBD-C18 with dimensions of 4.6 × 250 mm (i.d.) and a particle size of 5 μm (Agilent Technologies, Santa Clara, CA, USA). The mobile phase consisted of acetonitrile (A), alongside an aqueous solution of 0.3% phosphoric acid and 0.2% trimethylamine (B), in a volume ratio of 35:65. The flow rate was 0.7 mL/min, and the detector was set at a wavelength of 345 nm. The HPLC chromatograms of coptisine, palmatine, and berberine standards, along with alkaloids from the test sample, are presented in Figure S1.

The total alkaloid content was determined using a previously established method [16]. The test samples were diluted with methanol, and the absorbance of the resulting solutions was measured using a UV-Vis spectrophotometer (UV-1800, Shimadzu USA Manufacturing, Inc., Canby, OR, USA) at a wavelength of 345 nm. Standard berberine chloride was used to construct the calibration curve.

### 3.4. Extraction of Berberine, Palmatine, and Coptisine from C. chinensis

For the initial screening of the extraction solvents and methods, 0.2 g of *C. chinensis* powder was extracted with 4 mL of each respective solvent in a centrifuge tube, followed by vortex processing (250 VM vortex mixer, Hwashin, Seoul, Republic of Korea). The sample tubes were then kept in an ultrasonic instrument (S 100 H, Elma, Germany) at 50/60 Hz and a temperature of 30 °C for 30 min. Then, the resultant mixtures were centrifuged (Hermle Z 326 K, Hermle Labortechnik GmbH, Wehingen, Germany) at 4000 rpm for 15 min to remove solids. Subsequently, the extract solutions were diluted many times and filtered through a 0.45 μm membrane. Analysis was performed by HPLC, and each experiment was performed 3 times.

### 3.5. Response Surface Methodology

After the optimal solvent for berberine, palmatine, and coptisine extraction from *C. chinensis* was identified, efforts were made to investigate and optimize factors influencing extraction efficiency. Initially, individual factors, including solvent concentrations (ranging from 20% to 100%), liquid-to-solid ratios (varying between 10:1 and 30:1 mL/g), extraction times (ranging from 5 to 40 min), and extraction temperatures (ranging from 30 °C to 80 °C), were examined. Afterward, a Box–Behnken design (BBD) utilizing the RSM was employed to optimize the extraction parameters comprehensively. In this investigation, the experimental design incorporated the solvent concentration (A), liquid-to-solid ratio (B), extraction time (C), and extraction temperature (D) as independent variables, with berberine, palmatine, and coptisine extraction efficiency as the response variable. Each parameter was investigated at three levels, specified in Table 2, resulting in a total of 17 experimental runs comprising five central point assays within the study framework. Statistical analysis was conducted using Design-Expert 13.0 (Stat-Ease Inc., Minneapolis, MN, USA), with significance determined at a *p*-value less than 0.05.

### 3.6. The Recovery of Alkaloids from the Aqueous Extract

After the optimal parameters for the extraction of alkaloids from *C. chinensis* were identified, nine types of macroporous resins (HPD-300, HPD-400, DM-301, ADS-7, LSA-40, XAD-8, D-101, AB-8, and HP-20) purchased from Bon Adsorber Technology Co., Ltd., Cangzhou, China, were used to recover alkaloids from the extract. The experimental procedure began with preconditioning the columns, which entailed a sequential treatment involving absolute ethanol, 5% NaOH, and 5% HCl solutions. Following this treatment, the columns were rinsed thoroughly with deionized water until a neutral pH was attained. Subsequently, 5 mL of the extraction solution containing alkaloids, obtained under the optimized conditions, was passed through the prepared columns. After extraction, the columns were rinsed with deionized water to neutralize their pH. This process enabled the recovery of solvents, while alkaloids remained adsorbed in the columns. In the final phase, absolute ethanol was used to elute alkaloids, followed by the evaporation of ethanol to yield a product enriched with alkaloids. The recovery efficiency of alkaloids (REalka, %) was calculated using the following formula: REalka = (w/wo) × 100%, where ‘w’ denotes the quantity of alkaloids in the final product, and ‘wo’ denotes the quantity of alkaloids in the initial extraction solution.

### 3.7. Data Processing

An analysis of variance (ANOVA) was performed using SPSS version 20 software. To separate means, the least significant difference (LSD) test was applied with a significance threshold of *p* < 0.05. The statistical software Design-Expert (version 13.0, Stat-Ease Inc., Minneapolis, MN, USA) was utilized to optimize conditions.

## 4. Conclusions

A series of eco-friendly solvents, including aqueous solutions of surfactants, carboxylic acids, and their deep eutectic solvents, was produced to extract berberine, palmatine, coptisine, and total alkaloids from *C. chinensis*. In addition, an innovative method employing macroporous resins for the recovery of alkaloids from acid extracts was introduced. Lactic acid, malic acid, and pyruvic acid exhibited the highest potential extraction rate of alkaloids among the solvents evaluated. The optimal conditions for the extraction of alkaloids from *C. chinensis* were identified based on systematic experimentation and the RSM. The highest extraction efficiency of alkaloids was achieved under the following conditions: with lactic acid at a concentration of 96% (*w*/*w*), a liquid-to-solid ratio of 30.0 mL/g, and an extraction temperature of 60.0 °C; with malic acid at a concentration of 40.0% (*w*/*w*), a liquid-to-solid ratio of 30.0 mL/g, and an extraction temperature of 80 °C; and with pyruvic acid at a concentration of 88.0% (*w*/*w*), a liquid-to-solid ratio of 30.0 mL/g, and an extraction temperature of 75.0 °C. Significantly, the alkaloid extraction efficiency using lactic acid, malic acid, and pyruvic acid as solvents exceeded that achieved with conventional volatile organic solvents and inorganic acids and bases. Regarding recovery potential, the XAD-8 and AB-8 resins exhibited the highest recovery efficiencies for alkaloids from lactic acid solutions, while the LSA-40 resin showed superior performance for malic acid and pyruvic acid solutions. The use of aqueous carboxylic acid solutions for extracting berberine, palmatine, coptisine, and total alkaloids from medicinal plants represents a more sustainable alternative to traditional methods, offering an optimized process, safety, sustainability, cost-effectiveness, biodegradability, ecological compatibility, and enhanced efficiency. Furthermore, this study represents the first comprehensive investigation into the efficacy of carboxylic acids for extracting protoberberine-type alkaloids from *C. chinensis*.

## Figures and Tables

**Table 1:** Summary of solvents used for the initial screening of berberine, palmatine, and coptisine extraction from *Coptis chinensis* Franch.

Type	Solvent	Abbreviation	CAS Number	Source	Concentration (% *w*/*w*)
Volatile organic	Ethanol	EtOH	64-17-5	Macklin (Shanghai, China)	99.7
					75
					50
					25
	Methanol	MeOH	67-56-1	Macklin	99.5
					75
					50
					25
	Acetone	Ace	123-54-6	Macklin	99
					75
					50
					25
Inorganic acid, base	Limewater	Lim	1305-62-0	Macklin	-
	Sulfuric acid	Sul	7664-93-9	Xilong (Shantou, China)	0.4
	Water	Water	-	-	100
Acid carboxylic	Acetic acid	AA	64-19-7	Macklin	99.5
					50
	Lactic acid	LA	10326-41-7	Macklin	96
					50
	Tartaric acid	TA	147-71-7	Macklin	50
	Citric acid	CA	77-92-9	Macklin	50
	Malic acid	MA	6915-15-7	Macklin	50
	Pyruvic acid	PA	127-17-3	Macklin	98
					50
	Propionic acid	PPA	79-09-4	Macklin	99.5
					50
	Malonic acid	MLA	141-82-2	Macklin	50
	Glycolic acid	GA	79-14-1	Macklin	50
Surfactant	Tween—65	T65	9005-71-4	Macklin	5 mM
	Tween—85	T85	9005-70-3	Macklin	5 mM
	Tween—60	T60	9005-67-8	Macklin	5 mM
	Tween—80	T80		Macklin	5 mM
	Tween—40	T40	9005-66-7	Macklin	5 mM
	Tween—20	T20		Macklin	5 mM
	Triton—X—100	TX100	9002-93-1	Macklin	5 mM
	Triton—X—114	TX114	9036-19-5	Macklin	5 mM
	LAE—7	LAE-7	68439-50-9	Aseschem (Jodhpur, India)	5 mM
	LAE—9	LAE-9	68439-50-9	Novichem (Chorzów, Poland)	5 mM
	Brij—35	Brij-35	9002-92-0	Macklin	5 mM
	Choline chloride	-	67-48-1	Thermo Fisher Scientific (Waltham, MA, USA)	
DES	Choline chloride–Tartaric acid (1:1)	ChCl—TA (1:1)			100
	Choline chloride–Citric acid (1:1)	ChCl—CA (1:1)			100
	Choline chloride–Acetic acid (1:1)	ChCl—AA (1:1)			100
	Choline chloride–Lactic acid (1:1)	ChCl—LA (1:1)			100
	Choline chloride–Pyruvic acid (1:1)	ChCl—PA (1:1)			100
	Choline chloride–Glycerol (1:1)	ChCl—GL (1:1)			100
	Choline chloride–Propylene glycol (1:1)	ChCl—PG (1:1)			100
	Choline chloride–Ethylene glycol (1:1)	ChCl—EG (1:1)			100
	Glycerol		56-81-5	Macklin	
	Ethylene glycol		107-21-1	Macklin	
SUPRADES	Beta-cyclodextrin–Lactic acid (1:19)	β-CD-LA (1:19)			100
					50
	Beta-cyclodextrin–Pyruvic acid (1:19)	β-CD-PA (1:19)			100
					50
	Beta-cyclodextrin		7585-39-9	Macklin	

**Table 2:** The experimental design for the extraction of alkaloids from *Coptis chinensis*.

Variables	Coded Levels of Variables		
	−1	0	+1
Solvent concentration (%) (A)	20	60	100
Liquid–solid ratio (mL/g) (B)	10	20	30
Extraction time (min) (C)	5	22.5	40
Extraction temperature (°C) (D)	30	55	80

## Data Availability

The datasets generated and/or analyzed during the current study are available from the corresponding authors on reasonable request.

## Supplementary Materials

The following supporting information can be downloaded at: https://www.mdpi.com/article/10.3390/molecules30071418/s1, Table S1. Extraction efficiency of alkaloids from *Coptis chinensis* by solutions of organic solvents and inorganic acids, and bases. Table S2. Extraction efficiency of alkaloids from *Coptis chinensis* by solutions of surfactant. Table S3. Extraction efficiency of alkaloids from *Coptis chinensis* by solutions of carboxylic acid. Table S4. Extraction efficiency of alkaloids from *Coptis chinensis* by solutions of deep eutectic solvents and supramolecular deep eutectic solvents. Table S5. Extraction efficiency of alkaloids from *Coptis chinensis* using varying concentrations of lactic acid (A). pyruvic acid (B). and malic acid (C). Table S6. Extraction efficiency of alkaloids from *Coptis chinensis* using varying liquid-to-solid ratios of lactic acid. pyruvic acid. and malic acid for each sample. Table S7. Extraction efficiency of alkaloids from *Coptis chinensis* using different extraction times with lactic acid (A). pyruvic acid (B). and malic acid (C). Table S8. The extraction efficiency of alkaloids from *Coptis chinensis* using different extraction temperatures with lactic acid (A). pyruvic acid (B). and malic acid (C). Table S9. Results of analysis of variance (ANOVA) with lactic acid as the solvent. Table S10. Results of analysis of variance (ANOVA) with pyruvic acid as the solvent. Table S11. Results of analysis of variance (ANOVA) with malic acid as the solvent. Figure S1. HPLC chromatograms of coptisine, palmatine, and berberine standard (A), and alkaloids from test sample (B).
