# Supplementary material for: Alkaloid Extraction from Coptis chinensis Franch. Using Ultrasound-Assisted Aqueous Solutions of Surfactants, Organic Acids, Deep Eutectic Solvents, and Supramolecular Deep Eutectic Solvents
Source: Molecules. 2025 Mar 22;30(7):1418. doi: 10.3390/molecules30071418 (PMC11990161; doi:10.3390/molecules30071418)
Supplement: Supplementary file 1 [file molecules-30-01418-s001.zip › molecules-3504161-supplementary.pdf]

Supplementary material

Alkaloid Extraction from *Coptis chinensis* Franch. Using Ultrasound-Assisted Aqueous Solutions of Surfactants, Organic Acids, Deep Eutectic Solvents, and Supramolecular Deep Eutectic Solvents

Khan Viet Nguyen [1,†], Nhan Trong Le [1,†], Vy Thao Thi Dang [1], Oleh Koshovyi [2,*], Ain Raal [2] and Hoai Thi Nguyen [1,*]

[1] Faculty of Pharmacy, Hue University of Medicine and Pharmacy, Hue University, Hue City 49000, Vietnam; nvkhan@hueuni.edu.vn (K.V.N.); ltrongnhan@hueuni.edu.vn (N.T.L.); dangthithaovy92@gmail.com (V.T.T.D.)
[2] Institute of Pharmacy, Faculty of Medicine, University of Tartu, 50411 Tartu, Estonia; ain.raal@ut.ee
* Correspondence: oleh.koshovyi@ut.ee (O.K.); nthoai@hueuni.edu.vn (H.T.N.); Tel.: +380-509642706 (O.K.)
† These authors contributed equally to this work.

Supplementary material contents

- **Number of pages: 9**
- **Number of figures: 1**
- **Number of tables: 11**

Table S1. Extraction efficiency of alkaloids from *Coptis chinensis* by solutions of organic solvents and inorganic acids, and bases.

No.	Extraction solvent	Extraction efficiency (mg/g)			
		Coptisine	Palmitine	Berberine	Total alkaloids
1	MeOH 99.5%	4.8 ± 0.0	5.9 ± 0.1	35.5 ± 0.4	96.5 ± 0.4[e]
2	MeOH 75%	7.7 ± 0.0	7.1 ± 0.0	42.9 ± 0.1	108.2 ± 1.5[a]
3	MeOH 50%	7.3 ± 0.1	7.0 ± 0.1	41.4 ± 0.5	105.0 ± 0.5[c]
4	MeOH 25%	6.6 ± 0.3	6.1 ± 0.0	35.4 ± 0.2	87.8 ± 0.8[g]
5	EtOH 99.7%	1.8 ± 0.1	3.7 ± 0.1	21.9 ± 0.3	65.9 ± 1.0[h]
6	EtOH 75%	6.9 ± 0.1	7.0 ± 0.0	41.5 ± 0.3	107.9 ± 1.9[a.b]
7	EtOH 50%	7.8 ± 0.0	7.4 ± 0.1	43.8 ± 0.8	109.1 ± 0.9[a]
8	EtOH 25%	6.6 ± 0.2	6.8 ± 0.0	38.8 ± 0.1	91.9 ± 0.3[f]
9	Ace 99%	1.3 ± 0.2	3.0 ± 0.1	15.2 ± 0.6	28.3 ± 0.7[i]
10	Ace 75%	7.3 ± 0.1	7.8 ± 0.0	45.4 ± 0.3	100.3 ± 1.2[d]
11	Ace 50%	7.7 ± 0.2	6.3 ± 0.2	39.8 ± 1.0	79.8 ± 0.9[b.c]
12	Ace 25%	6.4 ± 0.1	6.1 ± 0.1	36.5 ± 0.5	93.1 ± 0.4[f]
13	$Ca(OH)_2$	4.6 ± 0.3	4.6 ± 0.2	26.81 ± 0.4	59.2 ± 0.7[l]
14	H_2SO_4 0,4%	8.5 ± 0.3	6.9 ± 0.1	42.47 ± 0.6	90.8 ± 0.3[j]
15	Water	4.3 ± 0.2	4.5 ± 0.2	26.71 ± 0.2	72.9 ± 1.3[k]

Different letters above each column indicate statistically significant differences ($p < 0.05$).

Table S2. Extraction efficiency of alkaloids from *Coptis chinensis* by solutions of surfactant.

No.	Extraction solvent	Extraction efficiency (mg/g)			
		Coptisine	Palmitine	Berberine	Coptisine
1	Tween – 65	5.6 ± 0.2	5.2 ± 0.2	31.3 ± 0.8	69.9 ± 2.4[c]
2	Tween – 85	5.4 ± 0.3	5.1 ± 0.2	30.5 ± 0.8	72.0 ± 1.7[b.c]
3	Tween – 60	4.9 ± 0.2	4.6 ± 0.1	28.0 ± 0.7	74.3 ± 0.4[a.b]
4	Tween – 80	7.1 ± 0.2	6.2 ± 0.1	36.8 ± 0.4	74.2 ± 2.3[a.b]
5	Tween – 40	4.6 ± 0.4	4.5 ± 0.3	27.6 ± 1.5	72.9 ± 2.3[b]
6	Tween – 20	5.0 ± 0.4	5.0 ± 0.1	29.8 ± 0.7	76.2 ± 1.6[a]
7	Triton – X – 100	4.5 ± 0.2	4.8 ± 0.1	28.1 ± 0.5	65.8 ± 2.1[d]
8	Triton – X – 114	4.4 ± 0.0	4.7 ± 0.0	27.9 ± 0.1	64.6 ± 1.1[d.e]
9	LAE – 7	4.2 ± 0.2	4.8 ± 0.2	28.3 ± 1.0	65.6 ± 1.5[d.e]
10	LAE – 9	4.4 ± 0.3	4.8 ± 0.1	28.3 ± 0.5	66.1 ± 0.7[d]
11	Brij – 35	4.6 ± 0.2	4.8 ± 0.0	28.4 ± 0.1	62.8 ± 0.8[e]

Different letters above each column indicate statistically significant differences ($p < 0.05$).

Table S3. Extraction efficiency of alkaloids from *Coptis chinensis* by solutions of carboxylic acid.

No.	Extraction solvent	Extraction efficiency (mg/g)			
		Coptisine	Palmitine	Berberine	Coptisine
1	LA 96%	9.9 ± 0.7	7.3 ± 0.5	45.0 ± 2.6	101.9 ± 1.9[e.f.g]
2	LA 50%	10.2 ± 0.4	7.6 ± 0.3	46.2 ± 1.3	111.7 ± 1.3[b]
3	AA 99.5%	7.4 ± 0.6	5.7 ± 0.2	35.8 ± 0.7	93.9 ± 2.1[h]
4	AA 50%	8.7 ± 0.4	6.5 ± 0.0	40.8 ± 0.1	109.9 ± 4.5[b.c.d]
5	TA 40%	8.2 ± 0.4	6.1 ± 0.1	38.4 ± 0.7	106.8 ± 0.1[c]
6	CA 50%	7.6 ± 0.1	5.5 ± 0.1	37.4 ± 0.5	101.3 ± 0.3[f.g]
7	PPA 99.5%	5.7 ± 0.4	4.9 ± 0.2	30.5 ± 1.2	99.9 ± 0.8[g]
8	PPA 50%	9.1 ± 0.2	6.8 ± 0.0	42.3 ± 0.1	105.6 ± 1.2[d.e]
9	MA 50%	9.8 ± 0.7	7.4 ± 0.5	45.1 ± 2.1	113.0 ± 1.4[b]
10	MLA 50%	8.2 ± 0.6	6.0 ± 0.0	38.1 ± 0.1	104.4 ± 1.1[d.e.f]
11	PA 98%	9.1 ± 0.4	6.9 ± 0.4	42.3 ± 1.0	119.8 ± 1.0[a]
12	PA 50%	10.2 ± 0.1	7.5 ± 0.1	46.2 ± 0.3	112.9 ± 2.7[b]
13	GA 50%	8.7 ± 0.1	6.6 ± 0.0	41.0 ± 0.2	92.2 ± 0.5[h]

Different letters above each column indicate statistically significant differences ($p < 0.05$).

Table S4. Extraction efficiency of alkaloids from *Coptis chinensis* by solutions of deep eutectic solvents and supramolecular deep eutectic solvents.

No.	Extraction solvent	Extraction efficiency (mg/g)			
		Coptisine	Palmitine	Berberine	Coptisine
1	ChCl – EG (1:1) 100%	5.3 ± 0.1	3.9 ± 0.1	26.9 ± 0.04	78.5 ± 0.12[f]
2	ChCl – PG (1:1) 100%	4.5 ± 0.1	3.4 ± 0.1	24.0 ± 0.03	65.1 ± 0.04[g]
3	ChCl – GL (1:1) 100%	4.2 ± 0.1	3.1 ± 0.0	22.7 ± 0.03	59.4 ± 0.05[h]
4	ChCl – CA (1:1) 100%	5.0 ± 0.2	3.7 ± 0.1	25.3 ± 0.04	81.2 ± 0.12[e.f]
5	ChCl – LA (1:1) 100%	6.2 ± 0.3	5.0 ± 0.1	31.9 ± 0.10	96.9 ± 0.02[d]
6	ChCl – TA (1:1) 100%	7.0 ± 0.5	5.5 ± 0.1	35.4 ± 0.06	96.8 ± 0.02[d]
7	ChCl – AA (1:1) 100%	7.0 ± 0.1	5.3 ± 0.0	33.7 ± 0.02	96.1 ± 0.02[d]
8	ChCl – PA (1:1) 100%	5.5 ± 0.1	4.3 ± 0.1	29.1 ± 0.03	82.3 ± 0.07[e]
9	β-CD – LA (1:19) 100%	8.0 ± 0.5	5.6 ± 0.4	37.1 ± 0.22	113.1 ± 0.16[a.b]
10	β-CD – LA (1:19) 50%	7.8 ± 0.4	5.6 ± 0.3	36.7 ± 0.15	107.1 ± 0.06[c]
11	β-CD – PA (1:19) 100%	7.9 ± 0.1	5.7 ± 0.2	37.5 ± 0.07	114.5 ± 0.31[a]
12	β-CD – PA (1:19) 50%	7.3 ± 0.3	5.1 ± 0.2	34.5 ± 0.11	110.4 ± 0.07[b]

Different letters above each column indicate statistically significant differences ($p < 0.05$).

Table S5. Extraction efficiency of alkaloids from *Coptis chinensis* using varying concentrations of lactic acid (A). pyruvic acid (B). and malic acid (C).

(A) - lactic acid

No.	Concentration of lactic acid	Extraction efficiency (mg/g)			
		Coptisine	Palmitine	Berberine	Total alkaloids
1	0	5.0 ± 0.1	4.8 ± 0.1	28.8 ± 0.4	66.3 ± 0.8[e]
2	20	8.7 ± 0.4	6.8 ± 0.2	42.2 ± 0.6	92.7 ± 1.0[d]
3	40	10.1 ± 0.2	7.5 ± 0.0	46.6 ± 0.4	100.7 ± 2.4[c]
4	60	10.6 ± 0.1	8.0 ± 0.0	49.1 ± 0.1	105.1 ± 1.3[a]
5	80	10.3 ± 0.1	7.5 ± 0.1	47.2 ± 0.3	104.4 ± 1.4[a.b]
6	96	9.8 ± 0.1	7.1 ± 0.1	45.0 ± 0.3	101.9 ± 1.9[b.c]

(B) - pyruvic acid

No.	Concentration of pyruvic acid	Extraction efficiency (mg/g)			
		Coptisine	Palmitine	Berberine	Total alkaloids
1	0	5.0 ± 0.1	4.8 ± 0.1	28.8 ± 0.4	66.3 ± 0.8[e]
2	20	9.9 ± 0.1	7.5 ± 0.0	46.1 ± 0.3	97.7 ± 0.6[d]
3	40	10.0 ± 0.1	7.5 ± 0.1	46.3 ± 0.2	104.1 ± 1.5[c]
4	60	10.3 ± 0.2	7.6 ± 0.1	47.3 ± 0.6	105.3 ± 1.6[b.c]
5	80	10.6 ± 0.3	7.7 ± 0.2	47.9 ± 0.9	108.2 ± 1.1[a.b]
6	98	10.8 ± 0.1	7.8 ± 0.1	48.7 ± 0.4	112.0 ± 1.5[a]

(C) - malic acid

No.	Concentration of malic acid	Extraction efficiency (mg/g)			
		Coptisine	Palmitine	Berberine	Total alkaloids
1	0	5.0 ± 0.1	4.8 ± 0.1	28.8 ± 0.4	66.3 ± 0.8[e]
2	10	8.7 ± 0.2	7.0 ± 0.2	42.8 ± 1.0	92.6 ± 1.3[d]
3	20	9.5 ± 0.2	7.4 ± 0.1	45.8 ± 0.4	96.0 ± 2.1[c]
4	30	9.9 ± 0.1	7.6 ± 0.1	46.8 ± 0.2	98.1 ± 0.9[b.c]
5	40	9.8 ± 0.1	7.5 ± 0.0	46.4 ± 0.2	100.3 ± 1.5[a.b]
6	50	10.3 ± 0.1	7.7 ± 0.1	47.7 ± 0.6	102.5 ± 1.8[a]

Different letters above each column indicate statistically significant differences ($p < 0.05$).

Table S6. Extraction efficiency of alkaloids from *Coptis chinensis* using varying liquid-to-solid ratios of lactic acid. pyruvic acid. and malic acid for each sample.

(A) - lactic acid

No.	Liquid-to-solid ratio (mL/g)	Extraction efficiency (mg/g)			
		Coptisine	Palmitine	Berberine	Total alkaloids
1	10	9.0 ± 0.1	7.2 ± 0.1	40.4 ± 0.4	86.4 ± 1.2[d]
2	15	10.2 ± 0.1	8.0 ± 0.1	46.6 ± 0.3	108.9 ± 3.2[c]
3	20	10.7 ± 0.1	8.2 ± 0.0	50.0 ± 0.3	112.8 ± 2.1[b.c]
4	25	10.5 ± 0.1	8.0 ± 0.1	51.0 ± 0.2	121.4 ± 9.4[a]
5	30	9.9 ± 0.2	7.4 ± 0.1	49.5 ± 0.8	119.6 ± 0.9[a.b]

(B) - pyruvic acid

No.	Liquid-to-solid ratio (mL/g)	Extraction efficiency (mg/g)			
		Coptisine	Palmitine	Berberine	Total alkaloids
1	10	10.1 ± 0.0	7.8 ± 0.0	43.7 ± 0.6	90.4 ± 1.5[c]
2	15	11.3 ± 0.0	8.5 ± 0.0	49.9 ± 0.3	115.9 ± 1.7[b]
3	20	10.8 ± 0.1	8.0 ± 0.1	49.3 ± 0.6	115.7 ± 1.8[b]
4	25	11.1 ± 0.1	8.1 ± 0.1	55.4 ± 0.2	126.9 ± 1.2[a]
5	30	11.4 ± 0.2	8.1 ± 0.0	53.9 ± 0.1	117.7 ± 0.9[b]

(C) - malic acid

No.	Liquid-to-solid ratio (mL/g)	Extraction efficiency (mg/g)			
		Coptisine	Palmitine	Berberine	Total alkaloids
1	10	8.7 ± 0.1	6.9 ± 0.1	38.5 ± 0.4	78.2 ± 1.0[d]
2	15	10.4 ± 0.1	8.0 ± 0.1	46.8 ± 0.5	96.9 ± 2.5[c]
3	20	10.2 ± 0.1	8.1 ± 0.1	49.6 ± 0.4	104.6 ± 3.6[b]
4	25	10.1 ± 0.1	8.0 ± 0.1	50.9 ± 0.4	114.6 ± 2.3[a]
5	30	9.8 ± 0.1	7.1 ± 0.0	48.6 ± 0.2	113.8 ± 1.8[a]

Different letters above each column indicate statistically significant differences ($p < 0.05$)

Table S7. Extraction efficiency of alkaloids from *Coptis chinensis* using different extraction times with lactic acid (A). pyruvic acid (B). and malic acid (C).

(A) - lactic acid

No.	Extraction time (min)	Extraction efficiency (mg/g)			
		Coptisine	Palmitine	Berberine	Total alkaloids
1	5	7.9 ± 0.2	6.2 ± 0.1	41.8 ± 0.6	124.0 ± 0.5[d]
2	10	9.7 ± 0.1	7.5 ± 0.1	48.5 ± 0.3	125.7 ± 1.4[c.d]
3	15	9.7 ± 0.2	7.5 ± 0.1	49.4 ± 0.8	119.3 ± 0.6[e]
4	20	9.8 ± 0.2	7.6 ± 0.1	50.4 ± 0.6	124.0 ± 2.4[d]
5	25	9.9 ± 0.0	7.7 ± 0.0	50.4 ± 0.1	126.9 ± 1.1[b.c]
6	30	10.1 ± 0.1	7.7 ± 0.1	50.8 ± 0.3	131.7 ± 1.3[a]
7	40	9.6 ± 0.1	7.4 ± 0.0	49.2 ± 0.1	127.7 ± 1.2[b]

(B) - pyruvic acid

No.	Extraction time (min)	Extraction efficiency (mg/g)			
		Coptisine	Palmitine	Berberine	Total alkaloids
1	5	10.0 ± 0.2	7.5 ± 0.2	49.4 ± 1.1	125.4 ± 1.7[e]
2	10	10.4 ± 0.1	7.8 ± 0.1	51.4 ± 0.6	133.3 ± 1.2[d]
3	15	11.0 ± 0.0	8.2 ± 0.0	54.0 ± 0.2	135.3 ± 1.5[c.d]
4	20	10.6 ± 0.1	8.0 ± 0.1	52.6 ± 0.4	136.7 ± 2.0[b.c]
5	25	11.0 ± 0.2	8.4 ± 0.1	54.3 ± 0.9	141.7 ± 1.3[a]
6	30	10.2 ± 0.1	7.8 ± 0.0	50.8 ± 0.3	139.4 ± 1.2[b]

(C) - malic acid

No.	Extraction time (min)	Extraction efficiency (mg/g)			
		Coptisine	Palmitine	Berberine	Total alkaloids
1	5	9.4 ± 0.0	7.2 ± 0.0	47.0 ± 0.2	113.9 ± 3.6[d]
2	10	8.5 ± 0.1	6.5 ± 0.1	42.9 ± 0.4	124.8 ± 1.4[c]
3	15	9.6 ± 0.1	7.3 ± 0.0	47.6 ± 0.4	132.0 ± 0.8[a]
4	20	10.0 ± 0.1	7.7 ± 0.0	49.6 ± 0.3	129.2 ± 0.8[b]
5	25	10.7 ± 0.1	8.2 ± 0.1	53.1 ± 0.6	124.1 ± 1.1[c]
6	30	10.0 ± 0.1	7.7 ± 0.1	50.0 ± 0.4	122.9 ± 0.5[c]

Different letters above each column indicate statistically significant differences ($p < 0.05$).

Table S8. The extraction efficiency of alkaloids from *Coptis chinensis* using different extraction temperatures with lactic acid (A). pyruvic acid (B). and malic acid (C).

(A) - lactic acid

No.	Extraction temperature	Extraction efficiency (mg/g)			
		**Coptisine**	**Palmitine**	**Berberine**	**Total alkaloids**
1	30	8.2 ± 0.0	6.4 ± 0.0	43.5 ± 0.2	116.8 ± 2.1[e]
2	40	8.1 ± 0.1	6.7 ± 0.0	45.4 ± 0.1	121.6 ± 3.0[d]
3	50	9.4 ± 0.2	7.3 ± 0.2	54.6 ± 0.4	126.6 ± 4.2[c]
4	60	9.0 ± 0.4	6.6 ± 0.2	50.1 ± 1.2	135.4 ± 1.9[a.b]
5	70	10.8 ± 0.1	8.0 ± 0.1	54.2 ± 0.7	138.0 ± 1.5[a]
6	80	9.5 ± 0.1	7.1 ± 0.1	47.7 ± 0.5	132.2 ± 0.6[b]

(B) - pyruvic acid

No.	Extraction temperature	Extraction efficiency (mg/g)			
		**Coptisine**	**Palmitine**	**Berberine**	**Total alkaloids**
1	30	8.7 ± 0.1	6.5 ± 0.0	44.5 ± 0.1	115.5 ± 1.8[d]
2	40	9.5 ± 0.0	7.5 ± 0.0	50.2 ± 0.1	123.3 ± 1.4[c]
3	50	10.1 ± 0.4	7.0 ± 0.2	52.4 ± 0.2	140.0 ± 1.0[b]
4	60	10.5 ± 0.1	7.6 ± 0.0	52.9 ± 0.6	139.7 ± 0.8[b]
5	70	10.9 ± 0.2	8.3 ± 0.1	54.7 ± 0.7	142.8 ± 0.4[a]
6	80	10.5 ± 0.1	7.9 ± 0.3	53.9 ± 0.2	139.7 ± 1.0[b]

(C) - malic acid

No.	Extraction temperature	Extraction efficiency (mg/g)			
		**Coptisine**	**Palmitine**	**Berberine**	**Total alkaloids**
1	30	8.3 ± 0.1	6.5 ± 0.1	43.9 ± 0.3	112.8 ± 1.4[d]
2	40	9.0 ± 0.0	7.2 ± 0.1	49.9 ± 0.3	124.1 ± 0.6[c]
3	50	9.3 ± 0.2	7.2 ± 0.1	47.8 ± 0.4	130.4 ± 1.1[a.b]
4	60	10.4 ± 0.1	7.3 ± 0.1	50.3 ± 0.1	131.9 ± 0.3[a]
5	70	10.1 ± 0.1	7.7 ± 0.1	50.9 ± 0.5	130.9 ± 0.9[a.b]
6	80	9.5 ± 0.0	7.3 ± 0.0	48.4 ± 0.2	129.5 ± 1.1[b]

Different letters above each column indicate statistically significant differences ($p < 0.05$).

Table S9. Results of analysis of variance (ANOVA) with lactic acid as the solvent.

Source	Sum of Squares	df	Mean Square	F-value	*p*-value	
Model	36.63	9	4.07	44.18	< 0.0001	significant
A	10.02	1	10.02	108.72	< 0.0001	
B	23.61	1	23.61	256.22	< 0.0001	
D	0.0683	1	0.0683	0.7414	0.4177	
AB	0.6878	1	0.6878	7.47	0.0292	
AD	0.0003	1	0.0003	0.0034	0.9554	
BD	0.0025	1	0.0025	0.0269	0.8745	
A^2	0.0024	1	0.0024	0.0256	0.8774	
B^2	2.23	1	2.23	24.15	0.0017	
D^2	0.0002	1	0.0002	0.0017	0.9685	
Residual	0.6449	7	0.0921			
Lack of Fit	0.3805	3	0.1268	1.92	0.2681	not significant
Pure Error	0.2644	4	0.0661			
Cor Total	37.28	16				
	R^2 = 0.9827		R^2 adj = 0.9605		R^2 pred = 0.8256	

Table S10. Results of analysis of variance (ANOVA) with pyruvic acid as the solvent.

Source	Sum of Squares	Df	Mean Square	F-value	*p*-value	
Model	41.40	9	4.60	74.11	< 0.0001	significant
A	6.27	1	6.27	101.01	< 0.0001	
B	23.87	1	23.87	384.57	< 0.0001	
D	8.01	1	8.01	129.11	< 0.0001	
AB	0.1319	1	0.1319	2.13	0.1882	
AD	0.3686	1	0.3686	5.94	0.0450	
BD	0.6380	1	0.6380	10.28	0.0149	
A^2	0.1807	1	0.1807	2.91	0.1317	
B^2	1.75	1	1.75	28.13	0.0011	
D^2	0.0583	1	0.0583	0.9396	0.3647	
Residual	0.4345	7	0.0621			
Lack of Fit	0.2105	3	0.0702	1.25	0.4021	not significant
Pure Error	0.2240	4	0.0560			
Cor Total	41.83	16				
	R^2 = 0.9896		R^2 adj = 0.9763		R^2 pred = 0.9111	

Table S11. Results of analysis of variance (ANOVA) with malic acid as the solvent

Source	Sum of Squares	df	Mean Square	F-value	*p*-value	
Model	37.31	9	4.15	111.71	< 0.0001	significant
A	1.97	1	1.97	53.12	0.0002	
B	26.92	1	26.92	725.47	< 0.0001	
D	3.16	1	3.16	85.10	< 0.0001	
AB	0.1263	1	0.1263	3.40	0.1076	
AD	0.0007	1	0.0007	0.0191	0.8940	
BD	0.0518	1	0.0518	1.39	0.2762	
A^2	1.44	1	1.44	38.84	0.0004	
B^2	3.38	1	3.38	91.01	< 0.0001	
D^2	0.0027	1	0.0027	0.0741	0.7934	
Residual	0.2598	7	0.0371			
Lack of Fit	0.0898	3	0.0299	0.7046	0.5972	not significant
Pure Error	0.1700	4	0.0425			
Cor Total	37.57	16				
	R^2 = 0.9931		R^2 adj = 0.9842			R^2 pred = 0.9547

(A)

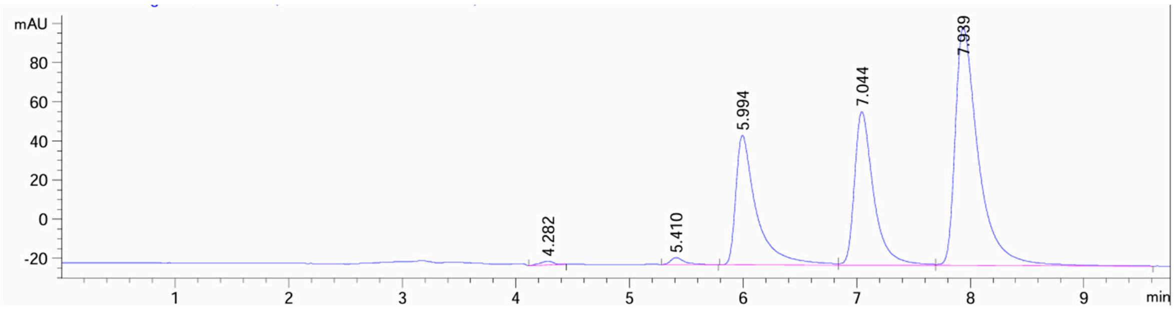

(B)

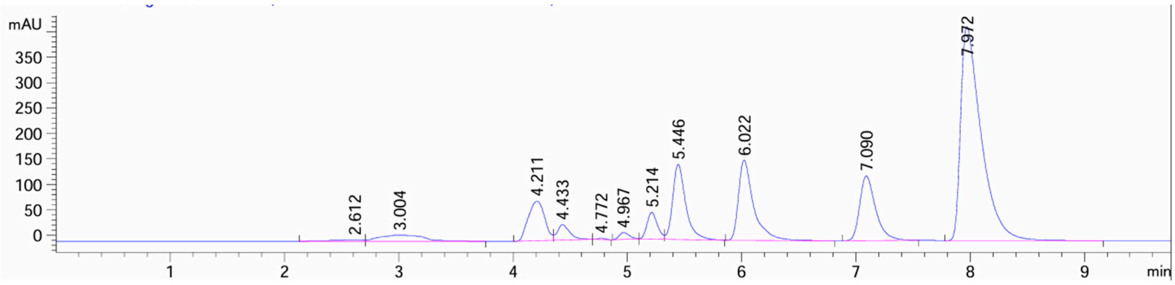

Figure S1. HPLC chromatograms of coptisine, palmatine, and berberine standard (A), and alkaloids from test sample (B).
